# Antibiotics and the developing intestinal microbiome, metabolome and inflammatory environment in a randomized trial of preterm infants

**DOI:** 10.1038/s41598-021-80982-6

**Published:** 2021-01-21

**Authors:** Jordan T. Russell, J. Lauren Ruoss, Diomel de la Cruz, Nan Li, Catalina Bazacliu, Laura Patton, Kelley Lobean McKinley, Timothy J. Garrett, Richard A. Polin, Eric W. Triplett, Josef Neu

**Affiliations:** 1 Department of Microbiology and Cell Science, Institute of Food and Agricultural Sciences, University of Florida, Gainesville, FL USA; 2 Division of Neonatology, Department of Pediatrics, University of Florida, Gainesville, FL USA; 3 Department of Pathology, Immunology and Laboratory Medicine, College of Medicine, University of Florida, Gainesville, FL USA; 4 Department of Pediatrics, College of Physicians and Surgeons, Columbia University, New York, NY USA

**Keywords:** Microbiology, Medical research

## Abstract

Antibiotic use in neonates can have detrimental effects on the developing gut microbiome, increasing the risk of morbidity. A majority of preterm neonates receive antibiotics after birth without clear evidence to guide this practice. Here microbiome, metabolomic, and immune marker results from the routine early antibiotic use in symptomatic preterm Neonates (REASON) study are presented. The REASON study is the first trial to randomize symptomatic preterm neonates to receive or not receive antibiotics in the first 48 h after birth. Using 16S rRNA sequencing of stool samples collected longitudinally for 91 neonates, the effect of such antibiotic use on microbiome diversity is assessed. The results illustrate that type of nutrition shapes the early infant gut microbiome. By integrating data for the gut microbiome, stool metabolites, stool immune markers, and inferred metabolic pathways, an association was discovered between *Veillonella* and the neurotransmitter gamma-aminobutyric acid (GABA). These results suggest early antibiotic use may impact the gut-brain axis with the potential for consequences in early life development, a finding that needs to be validated in a larger cohort.

*Trial Registration* This project is registered at clinicaltrials.gov under the name “Antibiotic ‘Dysbiosis’ in Preterm Infants” with trial number NCT02784821.

## Main

Premature infants are particularly susceptible to infections secondary to increased need for invasive procedures and immaturity of the immune system, skin, and gastrointestinal tract^1–3^. The early microbiome in premature infants often is dominated by a single bacterial genus which can vary from child to child^4^. Microbiome composition then becomes increasingly complex^5–8^. There is growing concern that risk factors for mortality may originate from underlying pathologies that could also be responsible for premature birth^9^. Symptoms of prematurity are difficult to discern from symptoms of infection which, compounded by the increased risk of infection, have led to most premature infants being exposed to antibiotics early in life^10–12^. Despite high mortality rates, the incidence of culture positive early onset sepsis (EOS) is relatively low, between 0.2–0.6%^13^. In the absence of a positive culture, a majority of preterm infants receive antibiotics immediately after birth based on maternal risk factors (e.g. intra-amniotic infection) or laboratory abnormalities [e.g. elevated serum C-reactive protein (CRP)] because of the risk of mortality^13^. Given the low incidence of culture-positive EOS in this population, it is possible that such high rates of antibiotic use are unnecessary and may increase morbidity in these infants^14^. Other morbidities in the neonatal intensive care unit (NICU) such as necrotizing enterocolitis (NEC) and late onset sepsis (LOS) also have high mortality rates and have been associated with prolonged antibiotic exposure^15,16^. Nevertheless, antibiotics remain the most commonly prescribed medication in the NICU^17,18^.

The gut microbiome comprises a highly volatile community composition early in life ^1–8,19^. Microbial colonization is influenced as early as birth by mode of delivery, and perhaps even in the uterine environment by maternal factors^20,21^. Not surprisingly, antibiotic use has been shown to also change the composition of the preterm gut community^22–25^. Furthermore, antibiotic use early in life has increasingly been associated with adverse outcomes both short- and long-term^26,27^. One possible consequence is the disruption of the gut-brain axis (GBA), which involves bi-directional transmission of bio-molecular signals between the gut microbiota and the nervous system^28^. Aberrations in the GBA have been associated with altered immune homeostasis, as well as psychiatric, behavioral and metabolic conditions in adulthood^29^. It is therefore imperative to determine if such high rates of antibiotic use in preterm infants is necessary, as it could have lifelong consequences on future health.

Randomized clinical trials have the advantage of controlling for many of the numerous covariates that could interfere with answering whether preemptive antibiotic use in preterm infants affects outcomes. The routine early antibiotic use in symptomatic preterm neonates (REASON) study is the first to randomize symptomatic premature infants to either receive or not receive antibiotics soon after birth. Previously reported results from this study demonstrate the feasibility of such a trial and that withholding antibiotics did not lead to a significant increase in neonatal mortality or morbidity^30^. By employing a multi-omic approach, this cohort also provides the unique opportunity to understand how antibiotic intervention perturbs the early life gut microbiome, metabolome, and inflammatory environment in ways that may be consequential to health and development.

## Results

### Cohort and study description

The study protocol was approved by the IRB at UF on 9/2016, enrollment occurred from 01/2017 to 01/2019. Ninety-one of the total 98 enrolled infants had stool samples collected. Seven infants had no samples due to early mortality. Eligible infants were enrolled into groups based on previously described enrollment criteria^30^: group A—antibiotics indicated (n = 28), group B—antibiotics not indicated (n = 11), and group C—eligible for randomization (n = 52). Twenty-six infants were from group C1 (antibiotics in first 48 h) and 14 infants were from group C2 and did not receive antibiotics 48 h after birth. For 12 infants (46%) randomized to group C2, antibiotics were prescribed in the first 48 h after birth upon clinical assessment, and these infants were placed in a separate analysis group C2Bailed. Furthermore, one infant in group B was changed (bailed) to receive antibiotics within 48 h after birth, however this infant was excluded from this analysis since comparisons to a single subject would not be reliable. Therefore, there are a total of 90 infants with stool samples analyzed across 5 enrollment groups, 2 of which did not receive antibiotics within 48 h after birth (groups B and C2) and 3 of which did receive antibiotics immediately after birth (groups A and C1) or sometime within 48 h after birth having been bailed (group C2Bailed). Neither sex (*p* = 0.352) nor mode of delivery (*p* = 0.227) were significantly different between groups using the chi-square test. Both weight (*p* value = 0.005) and gestational age (GA) (*p* value = 0.002) were significantly different between groups overall, with group A infants on average with lower GA and at lower birth weights. Neither birth weight nor GA were significantly different between the randomized subgroups (C1, C2, C2Bailed) by the Kruskal–Wallis test (*p* > 0.05). In order to control for the confounding effect of varying GAs between groups, samples for corrected GAs were used between 28 and 39 weeks, the window encompassing the most samples among enrollment groups. A summary of the infants in this analysis is provided (Table 1). Likewise, we summarize the types of antibiotics and the number of times antibiotics were prescribed by group (Supplementary Table S1). A full description of enrollment has been described previously^30^. There were no statistically significant adverse clinical events between the randomized groups.

**Table 1:** Summary of infant enrollment, covariates, and samples.

	Group A	Group B	Group C1	Group C2	Group C2Bailed
Total infants	28	11	26	14	12
Sex (M::F)	14::14	4::7	11::15	7::7	9::3
Delivery mode (V::C)	13::15	7::4	10::16	6::8	2::10
Gestational age range (median)	24–32 (28)	29–32 (32)	25–32 (29)	23–32 (29)	24–32 (28)
Maternal antibiotic exposure (Yes::No)	20::8	6::5	20::6	8::6	11::1
Birth weight range in grams (median)	695–2132 (1015)	1100–2770 (1820)	525–2425 (1240)	630–2116 (1223)	605–1667 (888)
Samples post-normalization	232	42	171	99	98
Average number of samples/infant (median, 1st quartile, 3rd quartile)	8.3 (9, 5, 11)	3.8 (3, 2, 5)	6.6 (6, 4, 8.75)	7.1 (6, 4.5, 10)	8.2 (9.5, 4.25, 11.25)
Number of infants with metabolomic samples	4	0	2	2	2
Number of metabolomic samples (samples per infant)	32 (8, 10, 5, 9)	0	17 (12, 5)	16 (6, 10)	25 (14, 11)
Number of infants with immune marker samples	7	0	5	3	3
Number of immune marker samples (samples per infant)	40 (7, 7, 1, 3, 5, 7, 10)	0	22 (12, 2, 2, 5, 1)	21 (5, 10, 6)	26 (13, 11, 2)

Summary of the number of enrolled infants per group used in this analysis and the number of infants changed from group C2 to C2Bailed. Enrollment groups are also summarized by infant sex (male::female), mode of delivery (vaginal::caesarean), gestational ages, maternal antibiotic exposure (yes::no) and birth weight ranges in grams. The number of infants and number of stool samples used in the metabolomics and immune marker analyses are listed.

Six hundred ninety-three stool samples were collected longitudinally for 91 of the total 98 enrolled infants. Stool data were not available for 7 infants due to early mortality. Sequencing data for 16S rRNA were obtained for 656 of those samples. After rarefying to an even sequencing depth of 10,000 reads per sample, 642 samples remained. Since GA is significantly different between groups, we chose to focus on corrected GA between weeks 28–39 because there were not enough samples among groups at younger and older timepoints to derive meaningful comparisons. Therefore, 522 stool samples remained within this corrected GA window (Supplementary Table S2). The aim for this analysis is to test the effects of randomization to antibiotics vs. no antibiotics on the development of the gut microbiome, metabolome and inflammatory environment using high-throughput 16S rRNA sequencing, quantitative PCR (qPCR), metabolomics, pathway inference, immune marker analysis and open-source statistical tools.

### Antibiotic use and trends in early gut microbiome diversity development

**Figure 1:**
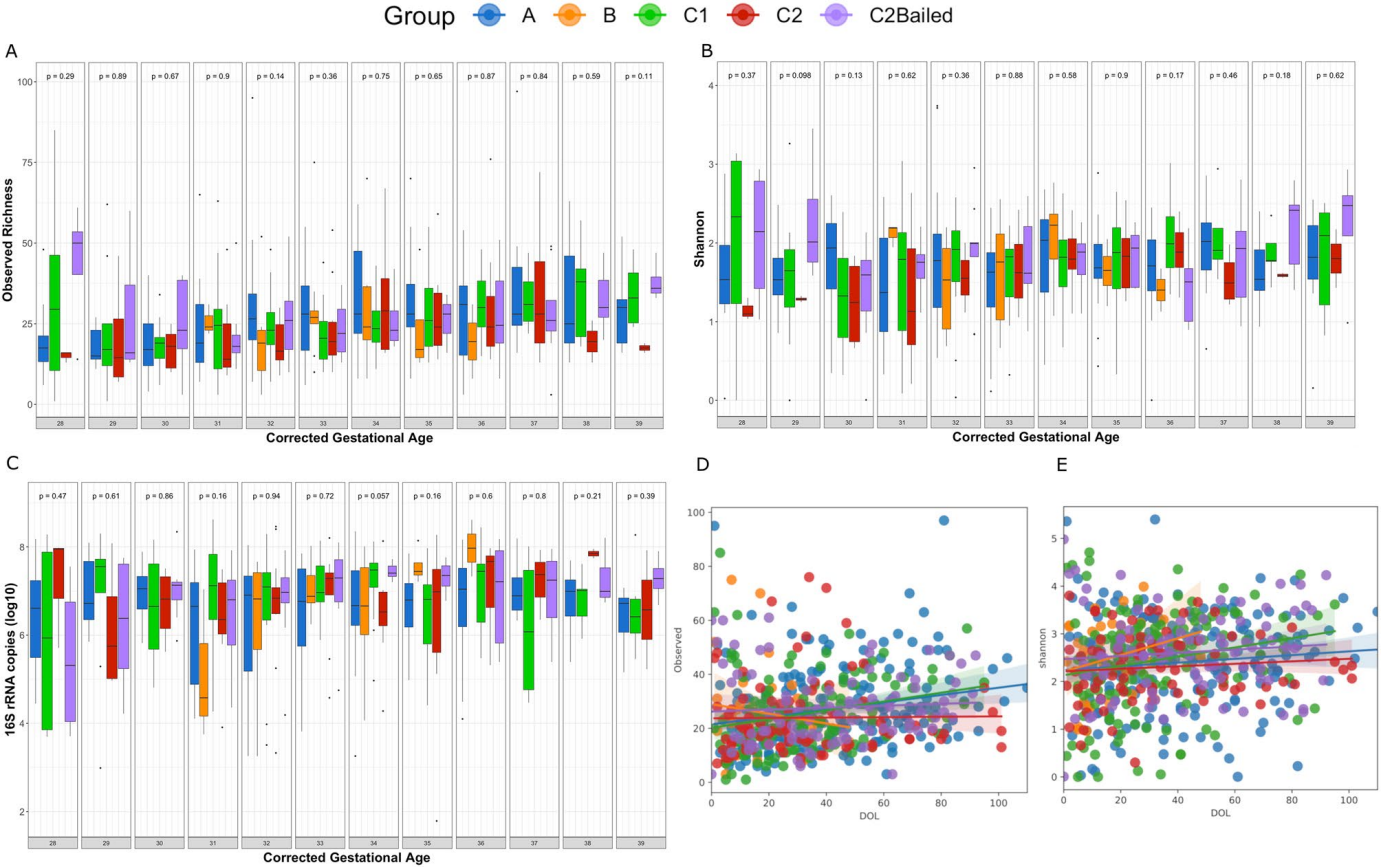
Antibiotic use 48 h after birth does not significantly affect alpha diversity development. Boxplots displaying (**A**) the observed ASV richness (**B**) the Shannon diversity and (**C**) log_10_-transformed copies of 16S rRNA by enrollment group across corrected gestational ages between weeks 28 and 39. *P* values were calculated at each corrected GA timepoint between enrollment groups using the non-parametric Kruskal–Wallis test. Linear mixed-effects modeling of the (**D**) observed ASV richness and (**E**) Shannon diversity over time between enrollment groups. Time scale on the x-axis is days of life (DOL) for corrected GA weeks 28–39. Greyed areas around each regression line represent 95% confidence intervals upper and lower around the coefficients.

Using amplicon sequencing variants (ASVs), the richness and Shannon alpha diversity were not significantly different between enrollment groups at each corrected GA timepoint using the Kruskal–Wallis test (Fig. 1A,B). Furthermore, the number of copies of 16S rRNA were not significantly different between groups at any timepoint (Fig. 1C). Interestingly, group B infants who did not receive antibiotics and were typically older and had an increasing trend in copies of 16S rRNA over time, but the same trend was weaker for group C2 infants who also did not receive antibiotics but were typically younger. Using a linear mixed-effects model (LME) through Qiime2^31–33^, neither richness nor Shannon diversity changed significantly over the time frame of corrected GA between 28–39 weeks (*p* = 0.407, *p* = 0.861, respectively) (Fig. 1D,E). Notably, groups C1 and C2Bailed had significant positive trends in richness over time (*p* = 0.019, *p* = 0.002, respectively). Groups B and C2 had negative trends in richness that were not significant (*p* > 0.05). All groups had positive trends in Shannon diversity development over time. However, none were significant (*p* > 0.05). Surprisingly, there was no significant difference in diversity between groups C1 (or similarly, C2Bailed) and C2, which are the informative groups for comparing effects of antibiotics or no antibiotics 48 h after birth. Certain considerations need to be made when comparing infants by enrollment group. For example, group A infants had significantly lower GA and birth weights, particularly compared to group B infants who on average had the highest GAs and birth weights, and the shortest stays in the NICU. Comparison between group B and the other enrollment groups is limited in scope because of shorter stays, i.e. fewer longitudinal samples. Furthermore, although the randomized groups C1 and C2 had similar number of enrolled infants in the beginning, nearly half of group C2 infants were bailed to receive antibiotics 48 h after birth. Therefore, the power to compare the randomized groups by antibiotic use with 48 h after birth is limited.

**Figure 2:**
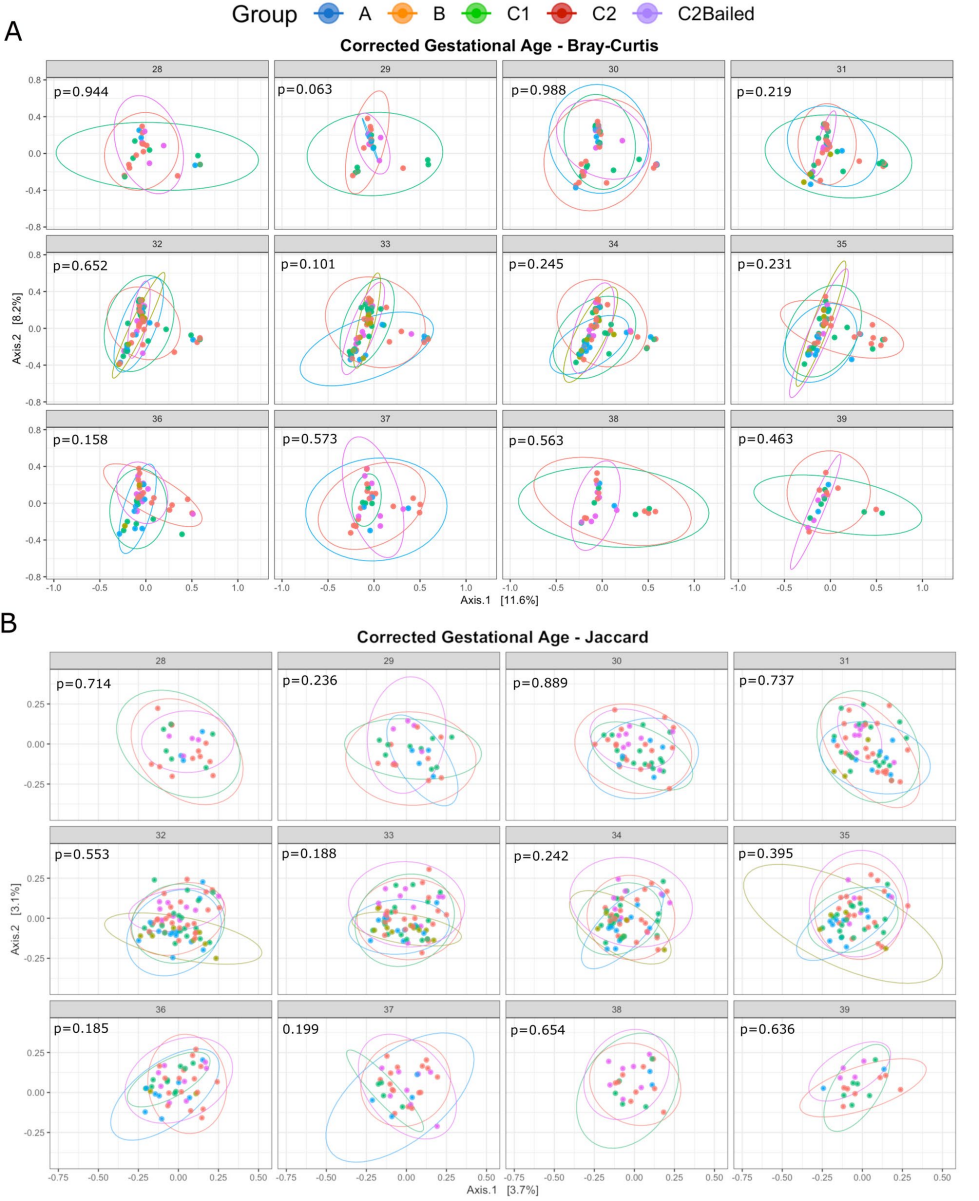
Antibiotic use explains little effect on beta diversity. PCoA ordination of stool samples using the (**A**) abundance-based Bray–Curtis and (**B**) presence/absence-based Jaccard distance metric among enrollment groups of corrected GA between 28–39. Ellipses are calculated based on a 95% confidence interval of a multivariate t-distribution.

Similar to alpha diversity, there were no apparent changes in overall bacterial community composition between groups when assessing beta-diversity over time and at each timepoint. Both the Bray–Curtis and Jaccard distance indices were used to assess community composition, taking into account quantitative ASV abundance and qualitative ASV presence/absence information, respectively. Principle coordinates analysis (PCoA) did not reveal any immediately obvious clustering differences between groups for either metric (Fig. 2), which may suggest little or no persistent overall effect of antibiotic use beyond 48 h after birth. Beta dispersion was not significantly different between groups using the Bray–Curtis metric (ANOVA; Df = 4, Sum of Squares = 0.011, Mean Squares = 0.003, F = 1.088, *p* = 0.362) but was significant using Jaccard (ANOVA: Df = 4, Sum of Squares = 0.048, Mean Squares = 0.012, F = 9.574, *p* = 1.79E−07), specifically group A versus all other groups (TukeyHSD, A vs. B: *p* = 0.010, A vs. C1: *p* value = 0.0003, A vs. C2: *p* = 0.004, A vs. C2Bailed: *p* = 0.00001). This might suggest differences in dispersion heterogeneity (i.e. greater spread in variance) between group A infants and infants in other groups, which could be explained by group A infants often receiving antibiotics beyond 48 h after birth. However, when the non-parametric permutational analysis of variance (PERMANOVA) test was applied to each timepoint across groups, there were no significant differences in Bray–Curtis or Jaccard distances among all groups at any given corrected GA timepoint (Fig. 2).

### Feeding patterns drive changes in gut diversity and bacterial load

**Figure 3:**
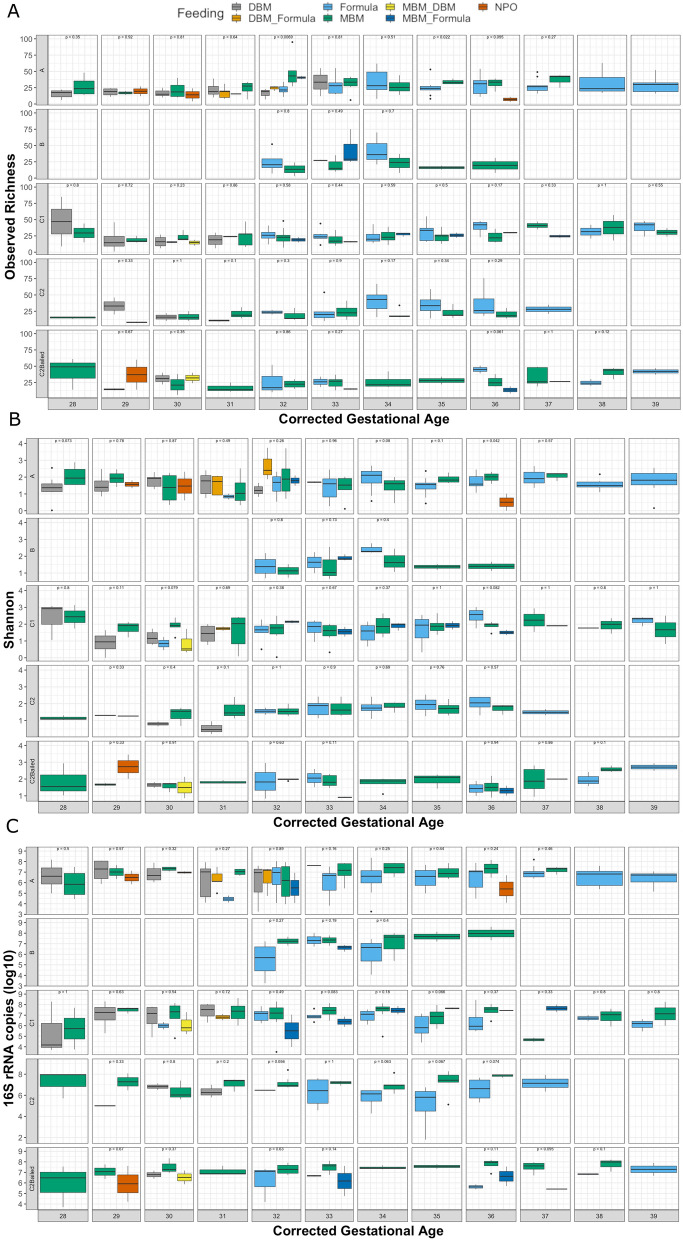
Effects of feeding patterns on the gut microbiome are transient over time. Effect of various feeding patterns on the (**A**) observed ASV richness (**B**) Shannon diversity and (**C**) log_10_-transformed copies of 16S rRNA by enrollment group over corrected GA from weeks 28–39. Only feeding patterns that have at least 2 samples at each timepoint were kept. Statistical comparison of feeding patterns at each corrected GA timepoint was performed using the non-parametric Kruskal–Wallis test.

For preterm infants, diet generally consists of mother’s breast milk (MBM), pasteurized donor breast milk (DBM), formula, or some combination of these sources. Notably, the formula used in this study did not contain specific probiotics or prebiotics. Some infants also experienced periods of no enteral feeding (NPO: nil per os). To investigate effects of feeding while still considering effects from antibiotic use, feeding types were compared within each respective analysis group. In addition, for purposes of comparing feeding types at each corrected GA timepoint, feeding types with only a single sample at each timepoint (n = 1) were removed. This reduced the total number of stool samples from 522 to 461. The number of samples in each group at each timepoint, and also by feeding type, is summarized in Supplementary Table S3. Since feeding was assessed on a week-by-week basis, previous feeding patterns are not indicative of the entire feeding history of the infant in a particular week. Using the calculated alpha diversity metrics described previously, feeding type was significantly different in bacterial richness only at corrected GA week 32 in group A infants (Kruskal–Wallis, *p* = 0.0069), where samples collected during feeding with all or partial mother’s milk tended to have higher bacterial richness (Fig. 3A). Furthermore, Shannon diversity was significantly lower in infants not fed orally at corrected GA week 36 in group A infants (*p* = 0.042) (Fig. 3B). The log_10_-transformed number of 16S rRNA copies were not significant at any timepoint in any group, although notably feeding with MBM alone typically had higher 16S rRNA copies compared with formula in all groups except for group A, which might be explained by continued antibiotic use beyond 48 h (Fig. 3C).

Beta diversity between feeding types within groups was likewise only significant at few specific timepoints. For group A infants, Bray–Curtis distances between formula and MBM feeding were significantly different at corrected GA week 34 using PERMANOVA (R^2^ = 0.093, *p* = 0.030). For group C1, Bray–Curtis distances were different between formula, MBM and MBM + formula at corrected GA week 36 (R^2^ = 0.370, *p* = 0.005). Also in group C1, Jaccard distances were significantly different between formula, MBM and MBM + formula at corrected GA week 34 (R^2^ = 0.141, *p* = 0.024), corrected GA week 35 (R^2^ = 0.158, *p* = 0.011) and corrected GA week 36 (R^2^ = 0.296, *p* = 0.007).

**Figure 4:**
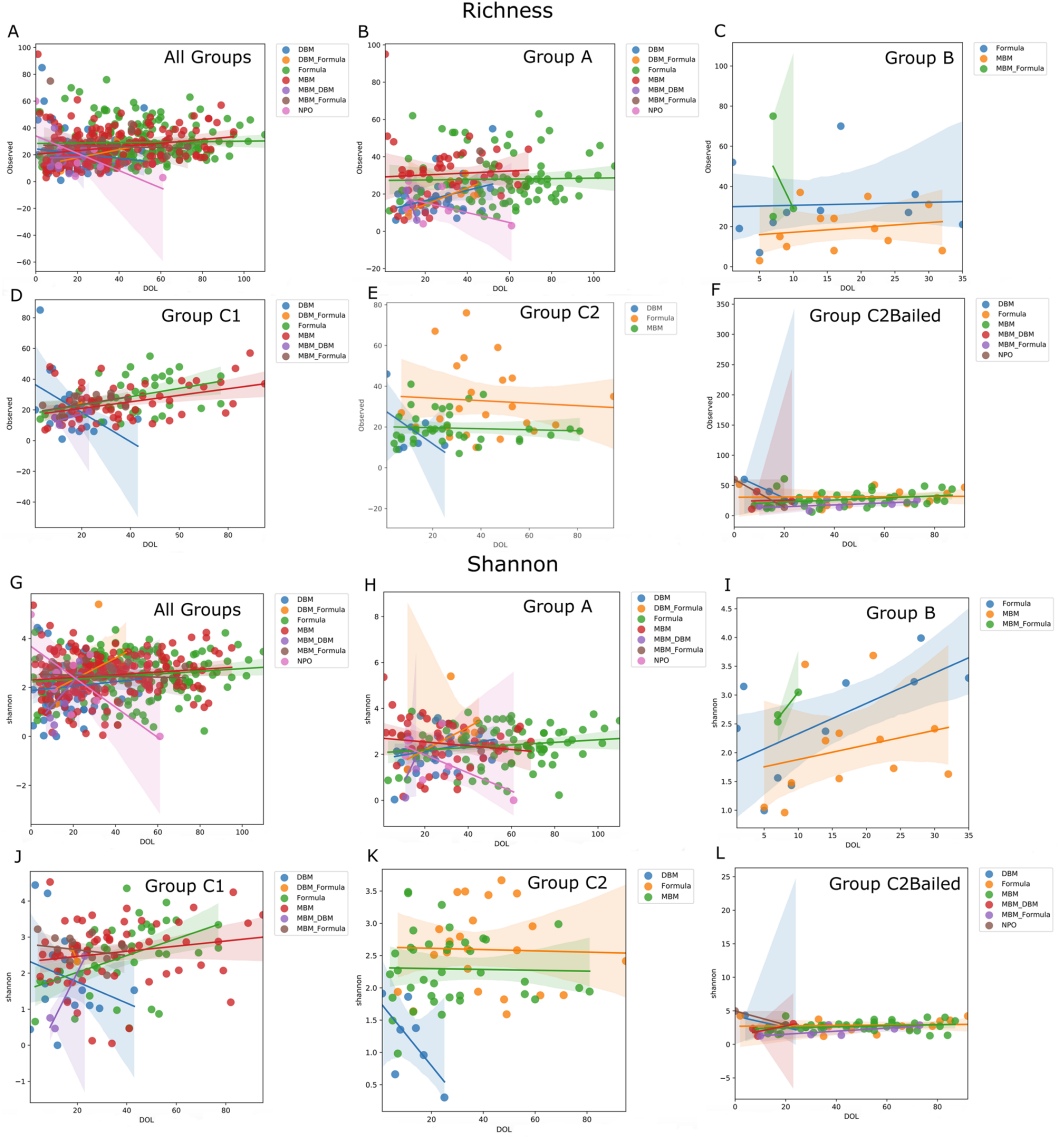
Linear mixed effects modelling identifies feeding effects by group over time. Linear mixed-effects results plotted as observed bacterial richness over time by group and feeding types (**A–F**) and the Shannon diversity over time by group and feeding types (**G–L**). The number of samples used in this analysis by group and by feeding type are listed in Supplementary Table S3.

Applying LME modelling by feeding type according to analysis group allows the observation of feeding effect over time, focusing again on the 12 weeks of corrected GA after removal of feeding type singletons at each timepoint (Fig. 4). Perhaps not surprisingly, among all groups, periods of NPO led to a lower trend in Shannon diversity over time (*p* = 0.003) (Fig. 4G). While bacterial richness appeared to trend lower, the trend was not significant (*p* = 0.341) (Fig. 4A). For group A infants, which received antibiotics in 48 h after birth and often beyond, MBM was associated with a slight increase in richness (*p* = 0.009) (Fig. 4B), and periods of NPO led to a lower trend in Shannon diversity (*p* = 0.031) (Fig. 4H). in Group B infants, who never received antibiotics and tended to be older, larger and healthier, all feeding types including formula (*p* = 0.004), MBM (*p* < 0.001) and MBM + formula (*p* = 0.018) led to increasing trends in Shannon diversity (Fig. 4I). However, no significant trends could be identified in richness (Fig. 4C). It is difficult to evaluate group B infants due to lower enrollment size, shorter NICU stays, and fewer samples overall. For the randomized infants that received antibiotics 48 h after birth, MBM and formula were associated with positive trends in richness (C1 and C2Bailed MBM: p < 0.001; C1 formula: *p* = 0.004; C2Bailed formula: *p* = 0.016) (Fig. 4D,F,J). Only group C2Bailed infants saw increased trends in Shannon diversity for feeding MBM (MBM only: *p* = 0.018; MBM + DBM: *p* = 0.004; MBM_formula: *p* = 0.002) (Fig. 4L). Finally, group C2 infants randomized to not receive antibiotics 48 h after birth saw a lower trend in both richness and Shannon diversity during feeding with DBM (Fig. 4E,K). However, neither trend was significant, likely due to the few numbers of samples. The power for detecting trends in group C2 is likely hampered because half of the infants randomized were bailed within 48 h after birth.

### Gut microbial community development is highly variable and unique to each infant

**Figure 5:**
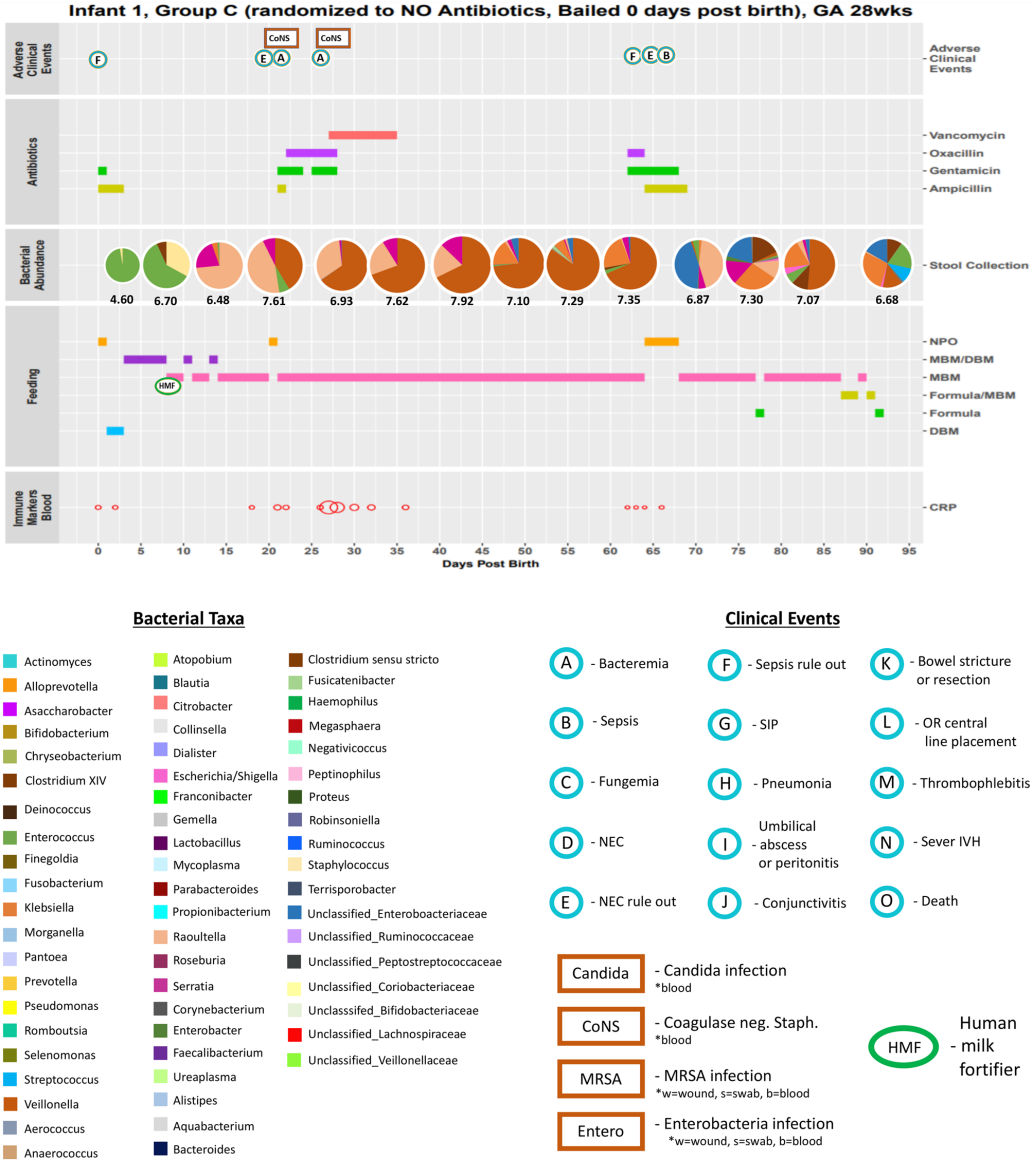
Integration of clinical and laboratory data gives detailed view of infant stay in NICU. Extensive clinical and laboratory data, when combined, provide a detailed summary of each infant’s stay in the NICU. Data included in each chart from top to bottom include: the infant ID, group assignment, antibiotic change status (bail), gestational age, any adverse clinical events (which are further described in Supplementary Figure S1), the type and duration of antibiotic use (if any), the copy-number corrected absolute composition of each weekly stool sample and it’s log_10_-scale number of bacterial 16S rRNA copies, the type and duration of each feeding including administration of human milk fortifier, the relative levels of C-reactive protein measured from blood, and relative concentrations of measured stool immune markers (for infants where these measurements were performed). DBM: donor breast milk, MBM: mother’s breast milk, NPO: no enteral nutrition, CRP: C-reactive protein, EGF: epidermal growth factor. Listed below is a key for the color-coded bacterial taxa used in the stool 16S rRNA copy-number corrected composition pie chart for each infant chart. A key for the bacterial color codes, adverse clinical events (including infections by body site) and the administration of human milk fortifier for each infant chart is given below.

Although the infants in this study were analyzed among 5 groups, each infant’s stay in the NICU is highly personalized, by type, frequency, number or length of antibiotic use, type and length of feeding patterns, and adverse clinical events over time. To aid in visual identification of patterns throughout the NICU course, detailed charts were created for each infant that depict both clinical and laboratory data over time (Days Post Birth) integrated into a single graphic per infant (Fig. 5 and Supplementary Fig. S1). This includes results from the 16S rRNA analysis as pie charts for each stool sample, color coded by bacterial taxonomy and sized based on the log_10_-transformed number of 16S rRNA copies per gram of stool, as well as adverse clinical events coded by a single letter code (Fig. 5). With these visualizations, patterns are more easily observed between antibiotic treatments, feeding types, and the gut microbiome. Typically, the microbiome composition after birth is homogenous in composition and diversifies, as well as increases in size over time, as might be expected. One interesting association involved the administration of the anti-fungal fluconazole and a resultant lowering of bacterial load and diversity. However, fluconazole was often administered in conjunction with antibiotics and was administered frequently to group A infants.

The more subtle effects of antibiotic use and feeding patterns become visually apparent by taking this individualized approach. To illustrate, infant 5 was exclusively fed mother’s milk from day 14 through day 71 post birth. At day 31, antibiotics vancomycin and piperacillin were administered (Supplementary Figure S1). During treatment, *Veillonella* was entirely removed from the stool microbiome, falling from 3.23E+06 cells per gram (73%) from the pre-treatment time point to an undetectable level while antibiotics were used. At day 47, 9 days after antibiotic treatment ceased, *Veillonella* again dominated the stool at 3.83 E+06 cells per gram (80%), almost a complete replacement of levels before treatment. Also, the proportions of the other 2 genera found in the stool, *Escherichia* and an unclassified Enterobacteriaceae spp., were nearly identical after treatment and continuation of mother’s milk as before treatment (pre-treatment: *Escherichia*—21%, Enterobacteriaceae spp. —4.7%; post-treatment: *Escherichia*—15%, Enterobacteriaceae spp. —3.3%). Thus, either that mother’s milk effectively restored the stool microbiome to its pre-treatment state or this effect occurred due to removal of antibiotic selective pressure, or both. A similar effect can be seen in infant 12 between 25 and 50 DOL where the microbiome is restored post-antibiotics. In this case, the restoration is observed with MBM, DBM, and formula. In some cases, antibiotic use had no effect on the microbiome composition (e.g. infant 42, 84), suggesting the presence of resistance mechanisms in the dominant gut microbes (in these 2 cases, members of Enterobacteriaceae). In fact, Enterobacteriaceae presence followed administration of ampicillin and gentamicin, the 2 most commonly prescribed antibiotics immediately after birth. This occurred in 24 of the 91 infants. Other times antibiotic use appears to dramatically and irreversibly change microbiome composition (e.g. infant 25).

### Bacterial genera correlate with stool metabolites and inferred metabolic pathways

**Figure 6:**
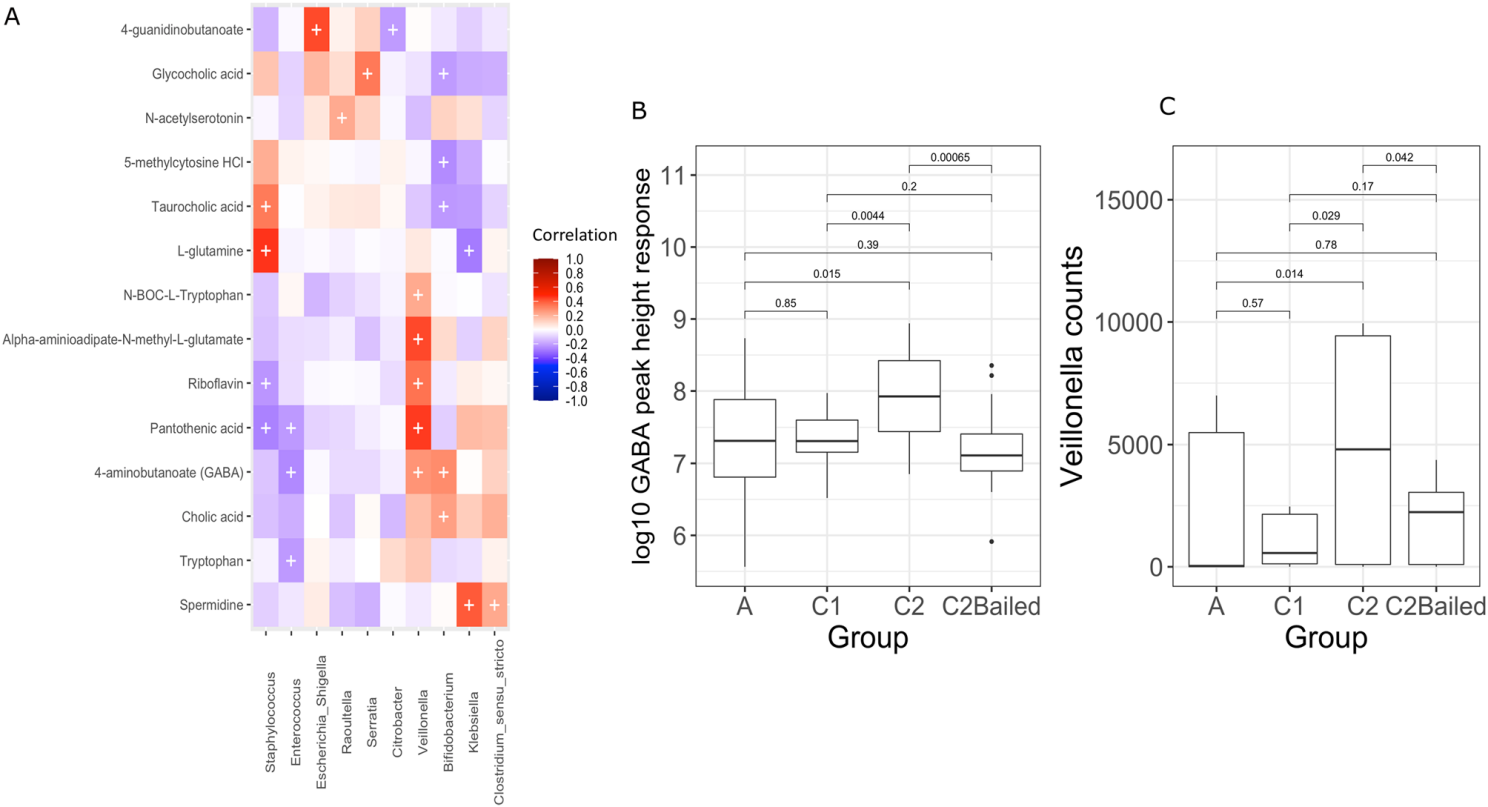
Metabolites in stool correlate with abundance of bacterial genera. (**A**) Heatmap of repeated measures correlation coefficients between peak response heights of identified metabolites in stool and the top 10 bacterial genera from the same samples (n = 90 stool samples). Significant correlations are indicated by a ‘ + ’ with FDR-corrected *p* values < 0.05. (**B**) Boxplot comparing the peak response heights for 4-aminobutyric acid (GABA) between enrollment groups. Statistical comparisons were made using the Wilcoxon test. (**C**) Boxplot comparing the number of rarefied *Veillonella* counts between the enrollment groups. Statistical comparisons were made using the Wilcoxon test. A summary of the number of infants and samples by group for metabolomics is given in Table 1.

In addition to 16S rRNA profiling, 90 stool samples from 10 infants were analyzed for metabolomic profiling (Table 1). Four of the 5 groups were included in these samples for comparison (group B samples not included). Peak height responses were recorded for 454 identifiable metabolites. To determine if gut bacteria were associated with relative concentrations of metabolites in stool, the top 10 most abundant bacterial genera associated with identified metabolites were determined. Repeated measures correlation values were plotted using a heatmap, which indicated numerous significant, positive and negative, associations between bacteria and metabolites (Fig. 6A). Interestingly, *Veillonella* were positively associated with the neurotransmitter 4-aminobutanoate (GABA) (R = 0.27, *p* = 0.013) and *Veillonella* counts were significantly different between groups A and C2 (*p* = 0.0475), C1 and C2 (*p* = 0.029), and C2 and C2Bailed (*p* = 0.042) using the Wilcoxon paired test (Fig. 6C). Also, *Veillonella* counts were not significantly different between samples of infants that received antibiotics, i.e. A and C1 (*p* = 0.57) or C1 and C2Bailed (*p* = 0.17). GABA peak height responses followed similar trends as *Veillonella* counts, that is, responses were significantly different between groups that received and did not receive antibiotics (A vs. C2, C1 vs. C2, C2 vs. C2Bailed) but not between groups that both received antibiotics (A vs. C1, C1 vs. C2Bailed) (Fig. 6B).

Furthermore, using PICRUSt2, functional pathway abundances were inferred based on the rarefied 16S rRNA data^34^. The *Veillonella* counts of predicted pathways were strongly correlated with biosynthesis of the GABA precursor L-glutamate (R = 0.88, *p* = 3.02E-27) (Supplementary Fig. S2). Thus, it may be that *Veillonella* could be at least partially responsible for GABA neurotransmitter production and that this function is negatively impacted by antibiotic use early in life. Alternatively, *Veillonella* may instead be involved in biosynthesis and export of L-glutamate in the gut, which is then converted to GABA by the host glutamate decarboxylase. However, PICRUSt2 results are based on inferred pathways from reference genomes closely related to the 16S rRNA data used here and are, at best, predictions in the absence of functional data specific to this cohort.

A negative correlation between *Bifidobacterium* counts and glycocholic acid was observed (R =  − 0.39, *p* = 0.0098), which was also impacted by antibiotic use between groups. In addition, bifidobacteria were negatively associated with other conjugated bile acids including taurocholic (R =  − 0.22, *p* = 0.045) and glycocholic acids (R =  − 0.21, *p* = 0.048), but positively associated with deconjugated cholic (R = 0.25, *p* = 0.027) (Fig. 6A). Thus, gut microbiota affected by antibiotic use may be responsible for modification of neuroactive metabolites (i.e. deconjugated bile salts) in addition to production of neurotransmitters.

### Immune markers in stool correlate with bacterial abundance

**Figure 7:**
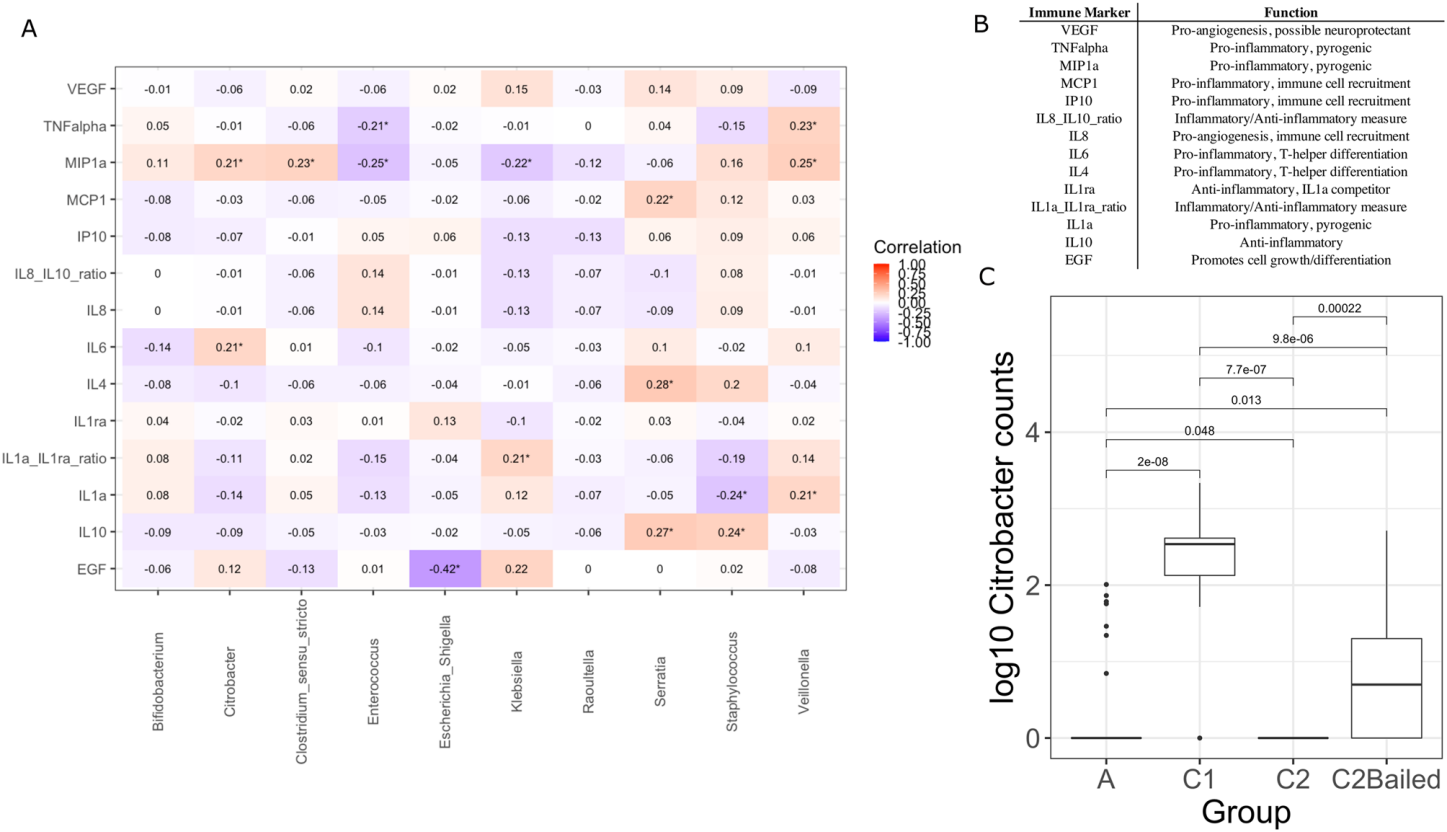
Stool immune marker levels show modest correlation with gut microbiota. (**A**) Heatmap of repeated measures correlation coefficients between immune markers measured from stool and the most abundant bacterial genera from the same samples (n = 110 stool samples). Only the bacterial genera with at least one significant correlation with an immune marker are displayed (10 genera). Significant correlations are marked with an ‘*’ by the coefficient, with FDR-adjusted p-values < 0.05. (**B**) Table listing the immune markers used for correlation analysis and their commonly known general functions. (**C**) Comparison of log_10_-transformed number of *Citrobacter* counts by enrollment group and their significance by the Wilcoxon test. A summary of the number of infants and samples by group for immune marker analysis is given in Table 1.

Antibiotic use was examined for its correlation with inflammatory marker levels in stool. These levels were also correlated with gut bacterial abundances. Twelve immune markers were measured in 110 stool samples across 18 of the first enrolled infants. A summary of immune marker data samples including infants per group and number of samples per infant is given in Table 1. Ten bacterial genera had at least one significant correlation with an immune marker (*p* < 0.05) (Fig. 7A). Significant correlations between the bacterial genera and stool immune markers were classified as either inflammatory or anti-inflammatory based on the known function of the marker (Fig. 7B). Interestingly, *Enterococcus* counts were negatively correlated with levels of TNF-alpha and macrophage inflammatory protein 1-alpha (MIP1α). *Citrobacter* were positively correlated with MIP1α and IL6 (R = 0.21, *p* = 3.74E−05), and were significantly higher in group C1 compared to group C2 (*p* = 7.7E−07) and group C2 compared to C2Bailed (*p* = 0.00022) by the Wilcoxon test (Fig. 7C). Lastly, counts of *Escherichia*/*Shigella* were significantly negatively correlated with levels of epidermal growth factor (EGF), which was the strongest correlation within the dataset. *Escherichia*/*Shigella* counts were highest among group A samples, but not significantly higher compared to other groups (Wilcoxon, *p* > 0.05).

## Discussion

The REASON study represents a significant step as it is the first randomized controlled trial to test the feasibility of randomizing symptomatic preterm infants to antibiotics versus no antibiotics, evaluating the effect of antibiotic treatment on the developing gut microbiome, metabolome, and inflammatory environment. Our results expand upon previous reports that early routine antibiotic use leads to alterations in the early life gut microbiome, even after discontinuation of antibiotics^22,35,36^. The results presented here suggest that antibiotic use 48 h after birth did not tend to have a lasting effect on the development of gut microbiome diversity over time, and that the gut microbiome diversity was recoverable. For example, alpha and beta diversity did not differ at each individual corrected gestational timepoint. Feeding patterns were also found to drive changes in the preterm gut microbiome, and each infant’s stay was highly unique to each infant. By combining metabolomics and immune signal profiling of the infant stool, specific microbe-metabolite and microbe-immune correlations were found, though more sampling is needed. The power to detect significant associations in this study was hampered mainly because many infants randomized to not receive antibiotics were changed to antibiotic administration. Furthermore, there were few infants who were enrolled in group B (an important antibiotic-free control group) and those enrolled in group B had few samples due to short stays in the NICU.

Our results support the notion that feeding types likely also have a significant influence on gut microbiome richness and diversity, though in this case only at specific timepoints^37–39^. Exclusive or partial feeding with mother’s own milk appeared to have higher bacterial load compared to formula and NPO, though not significantly. This observation is backed by previous evidence that breast milk harbors maternal-originating bacteria, as well as nutritional components (prebiotics) that support bacterial proliferation in the intestinal tract^40,41^. Interestingly, formula-fed infants had comparable levels of richness and diversity as mother’s milk. This supports the idea that mother’s milk drives early colonization of a limited set of dominant microbes through nutrient and antimicrobial-mediated selection^42–44^. Feeding trends over time were able to be assessed for the main feeding types such as MBM, DBM and formula however again the ability to detect meaningful results for rarer feeding types (particularly combinations of sources) suffered from few sample numbers.

Integrating detailed and personalized records of clinical and laboratory data led us to identified peculiar patterns, including that stool samples taken during administration of the anti-fungal fluconazole had lowered copies of 16S rRNA, suggesting lower bacterial load. A previous study reported that fluconazole, though not inherently bactericidal, increased the bactericidal activity of neutrophils^45^. Immune marker measurements in stool included calprotectin, which is secreted by neutrophils, and may help explain this pattern^46^. However, only 3 of the 18 infants that had immune marker data received fluconazole. Therefore, more data are needed to test this hypothesis. Interestingly, counts of *Enterococcus* were negatively correlated with levels of pro-inflammatory markers such as TNFα and MIP1α, an odd finding considering Enterococci have been associated with risk for infection in preterm neonates^47,48^. On the other hand, *Citrobacter* counts were associated with increased levels of the macrophage chemokine MIP1α and counts were significantly higher in infants randomized to receive or were bailed to receive antibiotics. Increased levels of MIP1α are likely related to recruitment of intestinal macrophages leading to a heightened inflammatory environment, suggesting that antibiotic use may select for bacteria which lead to intestinal inflammation^49^. Finally, *Escherichia*/*Shigella* counts had a relatively strong negative correlation with EGF levels. Previous studies have found that reduced concentrations of maternally derived EGF in mice correlated with *E. coli* gut translocation, and that supplementation with EGF protected the gut from colonization by enteropathogenic *E. coli* in a young rabbit model^50,51^. Perhaps EGF concentration could be important in ameliorating the effect of antibiotics on pathogen colonization in the preterm gut. Further work will be needed to understand how changes in the preterm infant gut caused by routine antibiotics impacts the gut inflammatory environment.

Neurological development can be impaired in infants born very prematurely compared to their full-term counterparts; a trend that extends into delayed cognitive and behavioral development through childhood^52–54^. Could routine early antibiotic use, or prolonged antibiotic use, in preterm neonates play a role in this association? Intestinal microbes produce a plethora of metabolites and bio-active compounds that can be absorbed by the host^55^. Some of these compounds have direct neurologic implications including neurotransmitters such as GABA, which is reduced in preterm infants, is critical for early brain development, and possesses immunomodulatory properties^56,57^. Antibiotic use negatively affected the abundance of *Veillonella* and that *Veillonella* were positively correlated with GABA concentrations in the gut. Furthermore, *Veillonella* correlated strongly with the L-glutamine biosynthesis pathway, the precursor to GABA production. *Veillonella* are also known for converting lactic acid to propionate, a short chain fatty acid which has been implicated in extracellular GABA accumulation by inhibiting the action of GABA transaminase, and this may also explain the positive correlation between *Veillonella* and GABA levels in the gut^58^. Aside from production of neurotransmitters, negative correlations were identified between *Bifidobacterium* abundance and concentrations of conjugated bile acids, particularly glyco- and taurocholic acid. Conjugated bile acids were also significantly different based on antibiotic use. Bifidobacteria, which were more abundant in infants that did not receive antibiotics, are known to deconjugate bile acids to primary forms including cholic acid^59,60^. Cholic acid can passively diffuse into the brain where it blocks signaling in the GABA_A_ receptor^61^. Bifidobacteria may therefore be essential in regulating GABA signaling in the developing brain. These are significant findings, for they suggest routine antibiotic use could be disrupting processes involved in the gut-brain axis and immunomodulatory pathways critical for neonatal and future childhood development.

Evidence-based antibiotic use to prevent infection in preterm neonates is critical in preventing unnecessary treatment that may be doing more harm than good. Overuse of antibiotics can change the developmental trajectory of the infant gut microbiome during a time of critical establishment and interaction. However, antibiotics remain a critical treatment for a population at greater risk for infection, and there naturally exists a delicate balance between when antibiotics are truly necessary for treatment or not. Given the potential for extensive crosstalk between gut microbiota and the host, changes in microbiome composition could have both short- and long-term effects on outcomes and overall health and development. This pilot study was largely limited by the relatively small number of enrolled infants, infrequent sampling once a week and reduced power since nearly half of the “no antibiotic” randomized infants were bailed. Future randomized studies with greater infant enrollment and more frequent sampling will be crucial in our understanding of the effects current neonatal practice has on health which will allow for the reevaluation of practices. Such trials will need to expand on the findings from this pilot study from a multi-omic standpoint to identify direct links between antibiotic-induced dysbiosis and health outcomes.

## Materials and methods

### Experimental design, enrollment, and clinical sample and data collection

The REASON study was conducted from January 2017–January 2019 at the University of Florida and was approved by the Institutional Review Board of the University of Florida (IRB201501045). The IRB-approved protocol is provided in the supplementary material. All methods were performed in accordance with the relevant guidelines and regulations. The Clinical Trial registration ID is NCT02784821. The registration date for this trial was May 27, 2016. All infants included in this study were enrolled after written informed consent was obtained from a parent and/or legal guardian. This study is funded by the NIH (R21HD088005). A detailed description of the study design including enrollment, group selection, randomization, and collection of clinical samples and data including outcomes has been previously described^25^. Infants born < 33 weeks gestation without major congenital anomalies, and admitted to the NICU, were eligible for enrollment. To summarize, 98 premature infants were enrolled in the study and placed into one of three groups according to previously described criteria: group A with indication for antibiotic use, group B without indication for antibiotic use, and group C eligible for randomization to antibiotics (C1) or no antibiotics (C2) in the first 48 h after birth. Infants not receiving antibiotics in the first 48 h after birth (group B, C2) could be changed to receive antibiotics at any time at the medical team’s discretion. There were no stewardship programs in place in the NICU during the study period. Furthermore, no infants enrolled in this study received probiotics during their time in the NICU. Clinical samples relevant to this analysis include weekly fecal collection starting with meconium when possible (all stored at − 80 °C) and results of bacterial and fungal cultures (blood, urine, sputum, and cerebrospinal fluid—when available) and laboratory measurements of CRP, white blood cell count and immature to neutrophil ratio. Clinical metadata from the mothers such as antepartum antibiotic use, type, duration, and proximity to delivery were recorded. Pertinent clinical metadata from the infants include group placement, antibiotic use status, antibiotics and antifungal use including type and duration throughout NICU course, feeding type and duration, GA at birth, sex, mode of delivery and any serious adverse events (SAEs) including NEC, late onset sepsis, spontaneous intestinal perforations, bronchopulmonary dysplasia, and death. The primary outcome was a composite serious adverse events; late onset sepsis, BPD, NEC, and death. Secondary outcomes were early onset sepsis intraventricular hemorrhage, periventricular leukomalacia, retinopathy of prematurity and spontaneous intestinal perforation. Sample size of 50 per group was planned to obtain sufficiently precise rates of anticipated admission to the NICU, consent, and estimates of clinical outcomes to plan a larger trial. This was subsequently changed to a goal enrollment for 100 infants based on admission rate. Block randomization was done by using random block sizes of two and four created in SAS. The input and results were uploaded into an electronic database used by the study team. Randomization group was disclosed to the study team and medical team.

### Stool DNA extraction, 16S rRNA PCR and sequencing analysis

DNA extraction and 16S rRNA barcoded PCR was carried out exactly as described previously^62^. Approximately 60 gigabases of nucleotide sequencing data was generated across 5 Illumina MiSeq flowcells for stool samples collected from 91 (of the 98 total) study participants where samples were collected (ICBR, Gainesville, FL, USA). The resulting sequencing reads were merged, demultiplexed, trimmed, filtered for quality and processed into amplicon sequencing variants (ASVs) as previously described with no alterations in method^62^. Briefly, sequences were processed to ASVs using the DADA2 package^63^ in R^64^ and assigned taxonomy using the SILVA_v132 training dataset^65–68^. Samples were rarefied to 10,000 reads per sample, leaving 642 of the total 656 individual longitudinal stool samples for analysis.

### Total bacterial quantification by universal 16S rRNA qPCR

Total bacterial load per gram of stool was determined by universal 16S rRNA qPCR using the same primer set used for amplicon sequencing (341F and 806R). QPCR assays were performed on a QuantStudio 3 system (Applied Biosystems, Life Technologies, USA). The reaction mixture contained 12.5 μl PowerUp SYBR Green 2X Master Mix (Applied Biosystems), 1 μl each of forward (341F) and reverse (806R) primer (10 μM), 1 μl of DNA template, 0.1 μg/μl BSA and brought to a final volume of 25 μl with nuclease free water. Cycling conditions were identical to those of the endpoint PCR used for sequencing. However, with a total of 40 cycles and replacing the final elongation step with a melt curve. Each sample reaction was performed in triplicate and these values were averaged for each sample copy calculation. A standard curve was generated for copy quantification using known concentrations of the expected PCR product amplified from a similar stool sample. Copies of 16S rRNA per gram of stool was calculated by multiplying the average copy number per replicate reaction (i.e. 1 μl DNA template) by the total DNA extraction volume (75 μl) and dividing this value by the mass of stool extracted in grams.

### Absolute bacterial abundance by copy number correction

Absolute bacterial abundance was calculated on a per gram of stool basis by correcting the relative sequencing abundance by the variable number of copies of the 16S rRNA gene in each observed organism. This correction was done using the “Estimate” tool provided as part of the rrnDB copy number database^69^. Briefly, after rarefying each sample to an even sequencing depth, the ASV sequences were submitted through the rrnDB online portal where they were classified down to the genus level using the RDP classifier version 2.12 and copy number adjusted using rrnDB copy number data version 5.6^69,70^. The copy number adjusted relative abundance for each observed taxon was multiplied by the total number of 16S rRNA copies obtained by qPCR, resulting in the absolute abundance of each taxon per gram of stool.

### Fecal inflammatory markers

Inflammatory markers were analyzed using a combination of multiplex technologies using the Bio-Rad Bio-Plex platform (Bio-Rad, California, USA). The markers evaluated include common markers of intestinal inflammation including calprotectin and S100A12, in addition to other markers such as IL-6, TNF, IL-10 and other cytokines and chemokines that may play a role in inflammatory or anti-inflammatory processes. The data were analyzed using direct comparisons of all infant groups using ANOVA and subsequent individual comparisons. Fecal calprotectin and S100A12 levels were measured using the fCal ELISA kit from BUHLMANN Laboratories AG (Schonenbuch, Switzerland) and the Inflamark S100A12 kit from Cisbio Bioassays (Codolet, France), respectively, according to the manufacturer’s instructions. Samples were then analyzed for the presence of both pro-inflammatory and anti-inflammatory cytokines/chemokines using Multiplex Human Cytokine Magnetic kit, Milliplex MAP Kit (Millipore, Billerica, MA, USA). Twelve cytokines/chemokines, including EGF, IL-10, IL-1RA, IL-B, IL-4, IL-6, IL-8, IP-10, MCP-1, MIP-1a, TNFα, and VEGF were analyzed according to the manufacturer’s instructions. Plates were read using the MILLIPLEX Analyzer 3.1 xPONEN System (Luminex 200). Cytokine concentrations were determined using BeadView software (Millipore, Billerica, MA, USA).

### Metabolomics

The infant stool samples were suspended in 400 µl 5 mM ammonium acetate. Homogenization was done 3 times for 30 s each time using a cell disruptor. Protein concentrations of the homogenates were measured using Qubit Protein Assay. Samples were normalized to 500 µg/ml protein at 25 µl for extraction. Each normalized sample was spiked with 5 µl of internal standards solution. Extraction of metabolites was performed by protein precipitation by adding 200 µl of extraction solution consisting of 8:1:1 acetonitrile: methanol: acetone to each sample. Samples were mixed thoroughly, incubated at 4 °C to allow protein precipitation, and centrifuged at 20,000 × g to pellet the protein. 190 µl supernatant was transferred into clean tube and dried using nitrogen. Samples were reconstituted with 25 µl of reconstitution solution consisting of injection standards, mixed, and incubated at 4° C for 10–15 min. Samples were centrifuged at 20,000 × g. Supernatants were transferred into LC-vials.

Global metabolomics profiling was performed as previously described using a Thermo Q-Exactive Orbitrap mass spectrometer with Dionex UHPLC and autosampler^71^. Briefly, samples were analyzed in positive and negative heated electrospray ionization with a mass resolution of 35,000 at m/z 200 as separate injections. Separation was achieved on an ACE 18-pfp 100 × 2.1 mm, 2 µm column with mobile phase A as 0.1% formic acid in water and mobile phase B as acetonitrile. The flow rate was 350 µl/min with a column temperature of 25 °C. Four µl was injected for negative ions and 2 µl for positive ions.

Data from positive and negative ions modes were processed separately. LC–MS files were first converted to open-format files (i.e. mzXML) using MSConvert from Proteowizard^72^. Mzmine was used to identify features, deisotope, align features and perform gap filling to fill in any features that may have been missed in the first alignment algorithm^73^. Features were matched with SECIM internal compound database to identify metabolites. All adducts and complexes were identified and removed from the data set prior to statistical analysis.

### Statistical analysis

The ASV and taxonomy tables resulting from DADA2 were manipulated using the phyloseq R package v1.30.0^74^. Inferred metabolic pathway abundances were determined from the rarefied 16S rRNA data using PICRUSt2^34^. Alpha diversity measures, including the observed number of ASVs and the Shannon diversity index were calculated using the microbiome R package v1.8.0 (https://bioconductor.org/packages/devel/bioc/html/microbiome.html). Box plots (including statistical testing where applicable) were generated using the ggpubr R package v0.2.4 (https://github.com/kassambara/ggpubr), which serves as a wrapper for ggplot2^75^. The linear mixed-effects modeling and associated plots were done using the longitudinal plugin “q2-longitudinal” offered in Qiime2 v2019.4^31–33^. The biomformat R package (https://biom-format.org) was used to convert data in phyloseq format to BIOM format for import into Qiime2^76^. Bray–Curtis and Jaccard distance dissimilarities were calculated using the vegan R package v2.5.6 (https://github.com/vegandevs/vegan) and PCoA plots were made using ggplot2 v3.3.0^75^. Individual infant charts were also generated using ggplot2^75^. Non-parametric statistical tests including the Wilcoxon and Kruskal–Wallis tests were used for pairwise and overall comparisons of 3 or more factors, respectively^77,78^. The permutational analysis of variance (PERMANOVA) test was used in the vegan package to compare overall microbiome dissimilarities between antibiotic use, feeding type, and enrollment groups. *p* values were adjusted for false discovery rate (FDR) via the Benjamin-Hochberg method^79^. Repeated measures correlation values (for non-independent repeated samples for multiple subjects) were calculated using the rmcorr R package^80^.

## Supplementary Information

Supplementary Figures.

Supplementary Figures.

Supplementary Information.

## Data Availability

The demultiplexed 16S rRNA sequencing data generated in this study are deposited in the NCBI Sequence Read Archive (SRA) under BioProject PRJNA515272.
