# Supplementary material for: Antibiotics and the developing intestinal microbiome, metabolome and inflammatory environment in a randomized trial of preterm infants
Source: Sci Rep. 2021 Jan 21;11:1943. doi: 10.1038/s41598-021-80982-6 (PMC7820285; doi:10.1038/s41598-021-80982-6)

**Antibiotics and the developing intestinal microbiome, metabolome and inflammatory environment in a randomized trial of preterm infants**

Jordan T. Russell^1†^, J. Lauren Ruoss^2†^, Diomel de la Cruz^2^, Nan Li^2^, Catalina Bazacliu^2^, Laura Patton^2^, Kelley Lobean McKinley^1^, Timothy J. Garrett^3^, Richard A. Polin^4^, Eric W. Triplett^1^*, and Josef Neu^2^

^1^Department of Microbiology and Cell Science, Institute of Food and Agricultural Sciences, University of Florida, Gainesville, FL, USA.

^2^Division of Neonatology, Department of Pediatrics, University of Florida, Gainesville, FL, USA.

^3^Department of Pathology, Immunology and Laboratory Medicine, College of Medicine, University of Florida, Gainesville, FL, USA.

^4^Department of Pediatrics, College of Physicians and Surgeons, Columbia University, New York, NY, USA.

*Corresponding author: Eric W. Triplett, PhD (email: ewt@ufl.edu)

†These authors contributed equally to this work.

**Supplementary Figure S1. Integrated clinical and laboratory data charts for all infants.** Data included in each chart from top to bottom include: the infant ID, group assignment, antibiotic change status (bail), gestational age, any adverse clinical events, the type and duration of antibiotic use (if any), the copy-number corrected absolute composition of each weekly stool sample and it’s log10-scale number of bacterial 16S rRNA copies, the type and duration of each feeding including administration of human milk fortifier, the relative levels of C-reactive protein measured from blood, and relative concentrations of measured stool immune markers (for infants where these measurements were performed). DBM: donor breast milk, MBM: mother’s breast milk, NPO: no enteral nutrition, CRP: C-reactive protein, EGF: epidermal growth factor. Collected stool samples that did not amplify for 16S rRNA during PCR are indicated by a black dot. Samples that were collected on or close to the same day are stacked, with the first collected sample on top.

**Supplemental Figure S2. Abundance of top bacterial genera correlate with abundances of inferred metabolic pathways**. Heatmap of repeated measures correlation values between counts of the 10 most highly abundant bacterial genera and abundance of inferred metabolic pathways using PICRUSt2. Only bacterial genera with at least one significant correlation are depicted (p-values < 0.05). n=90 stool samples.

**Supplementary Table S1.** Number and type of total antibiotics used by group and within the specified corrected GA analysis window. **Antibiotics given within 48 hours after birth

|  | Group A** | Group B  (1 bailed infant) | Group C1** | Group C2 | Group C2 Bailed** |
| --- | --- | --- | --- | --- | --- |
| # of days on antibiotics or antifungal (all routes) | 984 | 88 | 590 | 445 | 232 |
| # days of enteral/  parenteral antibiotics total | 771 | 59 | 503 | 368 | 212 |
| # days of enteral/  parenteral antibiotics x 28-39 weeks corrected GA | 476 | 14 | 479 | 272 | 179 |
| Antibiotics used | \| cefazolin \| \| --- \| \| ampicillin \| \| metronidazole \| \| clindamycin \| \| ceftriaxone \| \| gentamicin \| \| piperacillin-  tazobactam \| \| vancomycin \| \| oxacillin \| \| cefotaxime \| \| azithromycin \| \| ceftazidime \| \| cefoxitin \| \| meropenem \| \| fluconazole \| | \| ampicillin \| \| --- \| \| gentamicin \| \| vancomycin \| \| metroni-dazole \| | \| flagyl \| \| --- \| \| oxacillin \| \| cefotaxime \| \| acyclovir \| \| gentamicin \| \| metroni-dazole \| \| ampicillin \| \| vancomycin \| \| zosyn \| \| azithromycin \| \| tobramicyn \| \| cefepime \| \| fluconazole \| | \| vancomycin \| \| --- \| \| ampicillin \| \| oxacillin \| \| metroni-dazole \| \| penicillin \| \| gentamicin \| \| zosyn \| \| cefotaxime \| \| merem \| \| flagyl \| \| cefazolin \| \| azithromycin \| \| clindamycin \| \| cefepime \| | \| vanco-mycin \| \| --- \| \| cafazolin \| \| zosyn \| \| gentamicin \| \| ampicillin \| \| azithro-mycin \| \| oxacillin \| \| cefepime \| |

**Supplementary Table S2 – Samples for figure 1 by group and corrected gestational age.** The number of stool samples used in Figure 1 summarized by enrollment group and corrected gestational age.

|  | Group A | Group B | Group C1 | Group C2 | Group C2Bailed |
| --- | --- | --- | --- | --- | --- |
| Corrected GA 28 | 12 | 0 | 6 | 3 | 4 |
| Corrected GA 29 | 9 | 0 | 9 | 4 | 5 |
| Corrected GA 30 | 17 | 0 | 14 | 6 | 8 |
| Corrected GA 31 | 19 | 3 | 18 | 7 | 7 |
| Corrected GA 32 | 24 | 7 | 19 | 12 | 9 |
| Corrected GA 33 | 20 | 9 | 16 | 10 | 10 |
| Corrected GA 34 | 19 | 7 | 18 | 11 | 8 |
| Corrected GA 35 | 18 | 4 | 17 | 10 | 5 |
| Corrected GA 36 | 16 | 2 | 10 | 8 | 8 |
| Corrected GA 37 | 16 | 0 | 4 | 4 | 8 |
| Corrected GA 38 | 9 | 0 | 5 | 2 | 7 |
| Corrected GA 39 | 7 | 0 | 6 | 2 | 4 |

**Supplementary Table S3 - Samples for figures 2 and 3 by group, corrected gestational age and feeding type.** The number of stool samples used in Figure 2 summarized by enrollment group, corrected gestational age and feeding type. DBM: Donor Breast Milk; MBM: Mother’s Breast Milk; NPO: Nil Per Os (nothing by mouth).

|  | Group A | Group B | Group C1 | Group C2 | Group C2Bailed |
| --- | --- | --- | --- | --- | --- |
| Corrected GA 28 | 12 DBM: 8 MBM: 4 | 0 | 5 DBM: 3 MBM: 2 | 3 MBM: 3 | 3 MBM: 3 |
| Corrected GA 29 | 8 DBM: 4 MBM: 2 NPO: 2 | 0 | 7 DBM: 4 MBM: 3 | 4 DBM: 2 MBM: 2 | 4 MBM: 2 NPO: 2 |
| Corrected GA 30 | 15 DBM: 9 MBM: 4 NPO: 2 | 0 | 14 DBM: 4 Formula: 2 MBM: 5 MBM_DBM: 3 | 5 DBM: 2 MBM: 3 | 7 DBM: 2 MBM: 3 MBM_DBM: 2 |
| Corrected GA 31 | 18 DBM: 7 DBM_Formula: 5 Formula: 2 MBM: 4 | 0 | 15 DBM: 4 DBM_Formula:2  MBM: 9 | 6 DBM: 3 MBM: 3 | 3 MBM: 3 |
| Corrected GA 32 | 24 DBM: 3 DBM_Formula: 3 Formula: 10 MBM: 6 MBM_Formula:2 | 6 Formula: 4 MBM: 2 | 17 Formula: 6 MBM: 9 MBM_Formula: 2 | 9 Formula: 9 MBM: 7 | 7 Formula: 3 MBM: 4 |
| Corrected GA 33 | 19 DBM: 2 Formula: 11 MBM: 6 | 9 Formula: 3 MBM: 3 MBM_Formula: 3 | 16 Formula: 6 MBM: 8 MBM_Formula: 2 | 9 Formula: 5 MBM: 4 | 10 Formula: 2 MBM: 6 MBM_Formula: 2 |
| Corrected GA 34 | 19 Formula: 15 MBM: 4 | 6 Formula: 3 MBM: 3 | 18 Formula: 7 MBM: 8 MBM_Formula: 3 | 10 Formula: 5 MBM: 5 | 6 MBM: 6 |
| Corrected GA 35 | 15 Formula: 11 MBM: 4 | 2 MBM: 2 | 17 Formula: 8 MBM: 7 MBM_Formula: 2 | 10 Formula: 4 MBM: 6 | 3 MBM: 3 |
| Corrected GA 36 | 15 Formula: 9 MBM: 4 NPO: 2 | 2 MBM: 2 | 10 Formula: 4 MBM: 4 MBM_Formula: 2 | 8 Formula: 5 MBM: 3 | 8 Formula: 2 MBM: 4 MBM_Formula: 2 |
| Corrected GA 37 | 13 Formula: 10 MBM: 3 | 0 | 4 MBM: 2 MBM_Formula: 2 | 2 Formula: 2 | 7 MBM: 5 MBM_Formula: 2 |
| Corrected GA 38 | 6  Formula: 6 | 0 | 5  Formula: 2  MBM: 3 | 0 | 6  Formula: 3  MBM: 3 |
| Corrected GA 39 | 7 Formula: 7 | 0 | 5 Formula: 3 MBM: 2 | 0 | 2 Formula:2 |

# IRB-approved protocol for REASON: Institutional Review Board of the University of Florida no. IRB201501045.

# Antibiotic Effects on the Developing Microbiome, Metabolome and Morbidities in Preterm Neonates

**Specific Aims**

This research challenges existing dogma for pre-emptive antibiotic use in preterm infants immediately after birth. Antibiotic use, especially when repeated, induces a perturbation (“dysbiosis”) in gut microbiota that may not recover to the basal state.[^1-3^](#_ENREF_1) Antibiotic use increases the risk of subsequent disease and adverse outcomes. [^4-8^](#_ENREF_4) The dependence of the developing immune system on the intestinal microbiota is supported by emerging evidence from studies in animals demonstrating decreased resistance to subsequent disease with early exposure to antibiotics. [^9-11^](#_ENREF_9) A majority of preterm very low birthweight (VLBW) infants are exposed to antibiotics. [^12^](#_ENREF_12)Surveys from large databases in the US show that the rate of culture proven bacteremia in these infants at birth is only between 1-2 percent,[^12^](#_ENREF_12)^,^[^13^](#_ENREF_13) which is consistent with our data from 3 University of Florida Hospitals, where we routinely do blood cultures after birth, but could find only 9 of nearly 900 infants less than 1,250 grams over a 5 year period whose cultures were positive. When cultures are done on cord blood with increased volumes of blood, there is a higher yield of positive cultures, but these are largely *Ureaplasma urealyticum* and *Mycoplasma hominis*[^14^](#_ENREF_14), mircoorganisms that are not that are not sensitive to the usual routine antimicrobial regimens provided right after birth. A retrospective review of 50,0261 neonates across 127 neonatal intensive care units (NICUs) from California showed a forty-fold variation in NICU antibiotic prescribing practice with similar burdens of proven infection and mortality.[^15^](#_ENREF_15) Based on this review, a large number of preterm infants are thus subjected to a potentially harmful course of antibiotics that provides no clear benefit. There remains a major gap in our understanding of antibiotic-related intestinal microbial dysbiosis and how this may result in disease.

Our hypothesis is *that early and prolonged antibiotic use in preterm neonates has significant consequences on the developing intestinal microbiome, metabolome and host response, predisposing the neonate to various major morbidities.* It is possible that the effect of this widespread antibiotic use outweighs the potential benefits. We propose a randomized trial of pre-emptive antibiotics versus no-pre-emptive antibiotics in preterms born at <33 weeks’ gestation. The purpose of this research is to evaluate the risks and benefits of current practice to determine optimal levels of antibiotic use that protects the babies from infection with minimal effect on the microbiome and subsequent adverse outcomes related to overuse of antibiotics. We will take advantage of the unique expertise of our team and facilities to achieve the following aims:

**Aim 1.** In a prospective, randomized pilot study, test the effects of pre-emptive postnatal antibiotics on the microbiome, metabolome and inflammatory responses in the neonate during the NICU course. We hypothesize that antibiotic use results in alterations in the microbial ecology of the intestinal microbiome that lasts well beyond discontinuance of antibiotic use and that this in turn results in altered fecal metabolic and inflammatory profiles that are associated with a higher incidence of adverse outcomes (Aim 2) while these infants are in the neonatal intensive care unit. .

**Aim 2.** In this same prospective, randomized pilot study, test the effects of pre-emptive postnatal antibiotics on adverse outcomes in the neonate while in the NICU. We hypothesize that higher antibiotic use will not be associated with decreased early onset sepsis and will be associated with increased adverse outcomes including necrotizing enterocolitis, late onset sepsis and mortality.

**Impact:** Here we will provide new data on the functional consequences of the widespread use of antibiotics in care of infants born preterm using novel multi-omic and systems biology approaches that will provide a better understanding of how these relate to common morbidities in the NICU. Fulfilling the aims of this research should stimulate multicenter trials that could ultimately lead to beneficial changes in perinatal and neonatal intensive care.

- - 1. **Significance**

The use of routine antibiotics during pregnancy in order to avoid adverse maternal and neonatal outcomes is widespread despite a relative lack of evidence for efficacy.[^16^](#_ENREF_16) One exception to this has been intrapartum antimicrobial prophylaxis for Group B-Streptococcal disease. [^17^](#_ENREF_17) There are no studies showing efficacy of postpartum prophylaxis against early onset sepsis. Antibiotics are by far the most common drugs given to neonates in the neonatal intensive care unit (NICU).[^12^](#_ENREF_12) The cost of these practices in terms of antibiotic-induced harm has only recently been questioned.[^18-22^](#_ENREF_18) In the preterm neonate, the rationale to routinely and pre-emptively give these antibiotics shortly after birth is speculative rather than evidence based and includes the immature immune system of these infants, the possibility that preterm delivery may have been caused by infection in the mother, that the immaturity-related respiratory distress cannot be readily distinguished from infectious pneumonia and that antibiotic use is safe. This may be incorrect. The average length of treatment of this “standard of care” practice is between 5-7 days.[^4^](#_ENREF_4)^,^[^13^](#_ENREF_13) Recent studies have shown an association between duration of early antibiotic use with increased odds of developing NEC, [^4^](#_ENREF_4)^,^[^5^](#_ENREF_5)a disease with extremely high mortality and morbidity.[^23^](#_ENREF_23) We [^24-26^](#_ENREF_24)and others[^27^](#_ENREF_27)^,^[^28^](#_ENREF_28) have shown that NEC and late onset sepsis (LOS) are preceded by an intestinal dysbiosis, which may partially be due to antibiotic usage. There has also been a trend toward resistant microbes causing late onset sepsis in infants treated with an early course of antibiotics and the gut is a major source of entry into the bloodstream for microorganisms via translocation.[^28^](#_ENREF_28) It has been shown that the intestinal microbiome is significantly affected by the early use of antibiotics, and that this effect often persists after discontinuation of the antibiotic.[^2^](#_ENREF_2)^,^[^19^](#_ENREF_19)^,^[^21^](#_ENREF_21)^,^[^29^](#_ENREF_29) It is very concerning that infants who receive 5-7 days of antibiotics in their first week had increased relative abundance of *Enterobacteriacae* and low diversity in the second and third weeks after birth.[^29^](#_ENREF_29) These infants also experienced more NEC, sepsis and death than those not exposed. [^29^](#_ENREF_29)

These studies beg for a systematic study of the antibiotic effects on the microbiomes and metabolomes of the infant along with the effects on morbidity to better understand the consequences of these practices. We **hypothesize** *that early and prolonged antibiotic use in preterm neonates has significant consequences on the developing intestinal microbiome, metabolome and host response, predisposing the neonate to adverse outcomes.* Since the rationale for this possible abuse of antibiotics is based on tradition and fear rather than evidence, equipoise amongst neonatologists for a large randomized trial is currently lacking: better evidence to support and guide such a trial is clearly needed. We propose that a systems biology approach utilizing “multi-omic” data will begin to fill a major gap in our knowledge of the consequences of antibiotic use in these infants that will lead to changes in practice. The data gathered in this pilot study will clarify the consequences of such widespread antibiotic use, and will be highly significant for lending credence for a larger trial which could lead to a **paradigm shift** in clinical neonatology. Our long-term goal is the development of regimes of antibiotic use (and perhaps microbial therapeutic measures) that provide protection against infection while maintaining gut health through a diverse microbiome.

- - 1. **Innovation**
- First randomized study of the effects of withholding pre-emptive antibiotics from preterm infants.
- First comprehensive evaluation of the microbiome, metabolome and inflammatory response in relation to adverse outcomes in neonates exposed to routine versus non-preemptive antibiotics.
- Development and use of novel bioinformatics tools that link clinical complex clinical metadata to the microbiome and metabolomes of preterm infants and their mothers.

**III. APPROACH**

**Aim 1.** In a prospective, randomized pilot study, test the effects of pre-emptive postnatal antibiotics on the microbiome, metabolome and inflammatory responses in the neonate during the NICU course.

**Rationale.** We previously showed that providing maternal antibiotics resulted in a lower microbial diversity of meconium in preterm infants born less than 32 week’s gestation. [^30^](#_ENREF_30) Preterm infants whose meconium microbes are less diverse have a greater association with late onset sepsis. [^31^](#_ENREF_31) A single dose of intrapartum antibiotics in another study resulted in significant shifts of the microbiota in the infants most evident at 30 days after birth.[^19^](#_ENREF_19) A recent study in mice elegantly showed that antepartum antibiotics can alter the offspring’s postnatal intestinal microbiota, decrease neutrophil and macrophage production via an interleukin-17 mediated mechanism, which in turn contributed to increased susceptibility of antibiotic-exposed neonatal mice to *Escherichia coli* K1 and *Klebsiella pneumoniae* sepsis.[^10^](#_ENREF_10) A recent Cochrane review included a total of 14 studies randomizing 7,837 women. An increase in neonatal deaths was shown for infants of women receiving prophylactic antibiotics when compared with placebo. Comparing any antibiotics with placebo, a marginally non-statistically significant increase was shown in any functional impairment.[^32^](#_ENREF_32) In addition to the dysbiosis-inducing effects that antibiotics can cause in the pregnant mother, routine antibiotic use in the preterm infant requires further investigation.

Dysbiosis consists of one or a combination of several types: loss of keystone bacteria, loss of diversity, shifts in metabolic components and/or a bloom of pathogens that increases systemic inflammation. Preliminary studies by our group using Shannon Diversity Index on fecal samples obtained from infants less than 33 week’s gestational age show that those infants who received no antibiotics had considerably higher diversity at 6 weeks’ post-birth compared to those infants who received ampicillin and gentamicin for only 1 week or longer. This suggests a major effect of postnatal antibiotics on the microbiota and begs further studies evaluating the effects of pre-emptively used antibiotics on preventing sepsis and their potentially detrimental effects using a “meta-biome” approach [^33^](#_ENREF_33)

**Patients and samples.**

All studies will be done at University of Florida Children’s neonatal intensive care unit (NICU) under an institutional review board (IRB) approved protocol. Mothers will undergo usual clinical care as per obstetrics. We plan to enroll 185maternal subjects and 185 neonatal subjects (approximately half of those available) during the course of the first year of this study. Informed consent will be obtained prior to delivery or within the first hours after birth.

A telephonic consent will be utilized when mothers are not available to be consented in person. This includes consenting mothers of neonatal subjects that are born in an outside facility and then transferred to UFHealth Shands’ NICU. Most often, the mother is not transferred with the infant and in such cases, the mother will be contacted at the outside facility for consent.

Subject to availability, we will be collecting a cutaneous microbiome sample within 24 hours of collecting a stool sample. This addition will allow for comparisons between the development of the microbiome in both the skin and gut within the same subject. It will also allow us to explore the impact of antibiotic exposure on cutaneous microbial diversity and adverse outcomes including sepsis. Skin swabs are collected using sterile technique. A cotton tip applicator will be rubbed on the subject’s forearm, or other area of skin easily accessible, in a circular motion for 20 seconds and placed in a sterile transport tube. Additional swabs will be collected at each time point to include a control “air” swab and an isolette/crib swab. The air swab will be opened and allowed to rest on the sterile field during the procedures and then place into a sterile transport tube. The crib swab will be opened and the cotton tip applicator will be rubbed on the inside of the isolette on the same side as the subject’s forearm that is used for the study. All three swabs will be moistened with 2-3 drops of sterile saline (sodium chloride) prior to collection.

In the event of adverse outcomes, cultured pathogenic organisms will be compared to microbial profiles of the skin and intestine to explore which site was likely breached. The skin swabs will be collected within 24 hours of stool sample collection.

Maternal diet can affect the fetal microbiome. We would like to gather information about the mother’s typical diet. We will provide the mothers an optional dietary recall form. As available, infant meconium and subsequent fecal samples (at least once a week), gastric fluid (from an existing standard of care gastric tube), and one breast milk sample (obtained in the first week after birth from mothers who choose to breast feed) will be collected and analyzed using techniques described in the approach section of the application. Fecal samples and skin swabs will be placed in -80-degree C freezers as soon as possible.

In terms of the rationale for obtaining the breast milk samples, the NICU at the Shands Hospital only provides human milk to babies born <33 weeks gestation in the first few weeks after birth. This can consist of milk from babies' own mothers or donor milk that is bought from a milk bank. Donor milk is pasteurized and should not contain live microbes, whereas babies' own mothers milk is known to contain cultivable microbes that may have a beneficial effect on the baby's developing gastrointestinal tract via alteration of the intestinal microbiome. In this study we have the opportunity to track whether the microbes from the mother's milk may be found in the infant GI tract and whether providing baby's own mothers milk versus donor milk may be a confounding factor that alters the microbial profile of the babies receiving antibiotics or not receiving the antibiotics. We will thus do 16S sequencing analysis on the microbial profile of both donor and breast milk samples provided to the infants.

Valuable information can be obtained from all groups of babies <33 weeks’ gestation, so all will be enrolled and have their microbiome, metabolome, inflammatory mediators and clinical outcomes analyzed. All of the individual groups will be categorized in intent to treat or not treat. Clinicians need to have the option to change treatment based on safety concerns for the patients.

Our overarching goal is to establish the effects of early antibiotic use on the infant intestinal microbial ecology as well as the effects on intestinal microbial metabolite and inflammatory marker production and subsequent morbidities. Extensive metadata from both mothers and infants for the newly derived samples will be transferred from our hospital EPIC electronic hospital record system into the REDCAP System. An enrollment log linking the subject’s name with their unique identifier will be kept in a password-protected, UF server. All other subject PHI will be transferred from directly from EPIC to REDCap. In order for our data to be able to interface with data sets being used for similar studies, we will utilize a newly developed standardized template for clinical studies in preterm birth.

Subjects will be de-identified by assigning the mother and the neonate(s) dyad a unique identifier. REDCap will assign neonatal subjects a subject number upon group assignment in REDCap. Maternal subjects will use the same subject number but will be linked to the neonate’s ID.

After samples are collected, they will be labelled with the above mentioned identifier and kept in a freezer in Dr. Neu’s lab in HD 318. This lab is locked.

Covariates for the mother include:

- Antibiotic use, type of antibiotic, and duration of therapy
- Length of rupture of amniotic membranes
- Prior or current infections
- C-section versus vaginal delivery
- Other drug use such as antacids or steroids
- Dietary history
- Body habitus
- Placental pathology reports

Covariates for the neonate include:

- Feeding type (baby’s own mothers’ milk, donor milk, formula, fortification)
- Morbidities (retinopathy of prematurity (ROP), chronic lung disease (CLD), bronchopulmonary dysplasia (BPD), necrotizing Enterocolitis (NEC), spontaneous ileal perforations (SIP), intraventricular hemorrhage (IVH), periventricular leukomalacia (PVL), sepsis after the first three days of life, sepsis before the first 3 days of life, death)
- Length of time on parenteral nutrition
- Duration of use of central arterial or venous catheters
- Growth velocities
- Complete blood count, blood culture, C-reactive protein results,
- Antibiotic use, type of antibiotic, and duration of therapy
- Date of birth
- Gestational age

Additional Covariates for the skin microbiome include:

- Location of swab collection (left arm, right arm),
- Time of last meal/feeding
- Time of last bath
- Type of last bath including soap/wipe details (if recorded)
- Topical medications and lotions applied
- Time of last handling/kangaroo care
- Time of last 2 diaper changes
- Type of bed (isolette, crib, etc)
- Start/stop dates of isolette humidity

**Randomization:**

We hypothesize that antibiotics will affect the microbial taxonomy, metabolic and inflammatory milieu of the developing gastrointestinal tract, and that non-pre-emptive use will not result in increased sepsis. We will have 3 groups to compare: the antibiotic-indicated group, the antibiotic not indicated group, and the randomized group, which will consist of 2 arms, subjects randomized to antibiotic therapy and those randomized to withhold antibiotic therapy

The “Antibiotic Indicated Group” is one that we cannot justify randomizing because they are highly likely to be septic. This would represent about 15% of the patients in our NICU as per our recent survey over the past 2 years. Guidelines for treating patients will be derived from newly published review based on a recent NIH workshop. As an example, if the mother has a temperature >39 degrees C. and if there are other symptoms such as fetal tachycardia, purulent fluid from the cervical os, maternal white blood cell count >15,000 in the absence of corticosteroids, the infant will be treated with antibiotics.

The “Antibiotics Not Indicated Group” comprises babies who are at extremely low risk. This would represent about 20% of patients <33 weeks’ gestational age admitted to our NICU as per a recent survey over the past 2 years. These infants are ‘asymptomatic” showing no significant signs of respiratory distress or having other risk factors.

Most of the infants who are “symptomatic” with respiratory distress requiring respiratory support over this time in our NICU were started on pre-emptive antibiotic therapy. This represents approximately 65% of the infants in our NICU over the past 2 years and is the group that will be randomized into “receive antibiotics” or “do not receive antibiotic” arms. Group allocation will not be blinded. Standard open label UF Clinical Research Center methodology will be used for randomization.  Respiratory distress, apnea and bradycardia, feeding intolerance or other factors clearly associated with prematurity will not be exclusion criteria. The intent will be that no baby randomized to receive antibiotics (ampicillin and gentamicin) will be treated for more than 48 hours. If the baby is allocated to the no antibiotic group and shows symptoms of deterioration along with lab values (e.g., increasing CRP, abnormal CBC) that could be consistent with sepsis, the clinician can choose to “bail out” and initiate antibiotics.

It is strongly suggested that all of these enrolled infants have blood cultures, complete blood counts and C-reactive proteins done shortly after birth as per routine care in our NICU. Infants whose blood cultures are positive at any time will have intravenous antibiotics initiated as per clinical team discretion.

The following scheme will be used for patient enrollment:

**Schedule of events:**

### Prenatally:

ICF is obtained from mother. This will be our primary means to obtain consent.

0-6 Hours after birth:

Informed consent obtained if not done prenatally and mother is able to give informed consent

- Infant is assigned to group A, B, or C based on obstetrical history and clinical appearance of subject. Subjects assigned to group C will be randomized to pre-emptive antibiotics or no pre-emptive antibiotics
- Obtain gastric aspirate from standard of care gastric decompression tube, if gastric fluid is present

### 1-3 days after birth:

- Collect meconium sample, if available
- Obtain 3ml of breast milk or donor milk if available
- Collect skin swab

Every week until neonate’s discharge:

- Collect stool samples, if available
- Collect skin swabs..

Approximately 60% of the mothers delivering babies who are born <33 weeks’ gestation at UF receive prenatal antibiotics and 80 % of these infants begin to receive antibiotics starting in the first 12 hours after birth. The usual postnatal antibiotic regimen (>95%) in consists of intravenously administered ampicillin and gentamicin. In order for our data to be able to interface with data sets being used for similar studies, we will utilize a newly developed standardized template for clinical studies in preterm birth[^34^](#_ENREF_34).

Inclusion Criteria: Valuable information can be obtained from all groups of babies <33 weeks’ gestation, so all consented neonatal subjects will have their microbiome, metabolome, inflammatory mediators and clinical covariates analyzed. All of the individual groups will be categorized in intent to treat or not treat. Clinicians need to have the option to change treatment based on safety concerns for the patients.

Exclusion Criteria: The only exclusions will be those from whom we are not able to obtain informed consent and those infants likely considered to be non-viable. Respiratory distress, apnea and bradycardia, feeding intolerance or other factors clearly associated with prematurity will not be exclusion criteria.

It will be strongly suggested that all enrolled infants will have blood cultures, complete blood counts and C-reactive proteins done shortly after birth as per routine care in our NICU. Infants in the “Antibiotics Not Indicated Group” and those “symptomatic” infants randomized to “do not receive antibiotics” whose blood cultures are positive at any time will have intravenous antibiotics initiated as per clinical team discretion.

For additional clarification on the length of time the antibiotics will be provided and the risks and benefits of the antibiotics, please note the following:

a) Statistics from the literature suggest that the average number of days preterm babies undergo treatment with antibiotics after birth is 5-7 days. This varies tremendously between different NICUs, with many trying to limit antibiotic use to 48 hours and in some cases 24-48 hours. In our protocol, the group that will likely undergo this length of treatment (5-7 days) is the first group where the suspicion for early onset sepsis is very high. However, in the group that is randomized, the length of time the babies randomized to receive antibiotics will be treated is projected to 48 hours. This 48 hour course is consistent with current practice in our NICU when the level of suspicion is actually low, but the babies are still given a course of antibiotics pre-emptively until cultures can be read and the results returned to the physician. Thus, the randomized group will actually not be undergoing an alternative treatment.
b) The risks of the antibiotics include: 1) the emergence of resistant microbes that may be much more difficult to treat;2) a higher risk of adverse outcomes such as increased necrotizing enterocolitis, late onset sepsis with more resistant organisms; 3) antibiotic induced metabolic alterations and epigenetic effects that may lead to diseases such as obesity in later life. The risk of not giving the antibiotic is that the baby may be harboring microbes that could cause an infection that is not being treated. We do not know whether the risks outweigh the benefits and that is the purpose of this study.
c) Occasionally a regimen of antibiotics other than ampicillin and gentamicin is chosen, usually for suspicion of renal failure risk or an infection that may be incurred by a microbe that is not sensitive to ampicillin and/or gentamicin. This occurs relatively seldom, and we will only use babies who receive this regimen in the randomized part of the study.

**Risks**:

The are no risks to the mothers who are enrolled in this study.

There is a potential risk that a neonate who is randomized to not receive antibiotics or in the “antibiotic not indicated group” could develop an infection. The primary medical team has the option to “bail out” of the randomization and initiate antibiotic therapy if the neonate shows signs of clinical deterioration along with corresponding lab values that support a presumptive diagnosis of sepsis (increasing C reactive protein, abnormal complete blood count values). Infants randomized to not receive antibiotics will receive a standard of care sepsis rule-out (CBC with differential, CRP, and blood cultures).

**Benefits:**

There are no benefits to the mothers who are enrolled in this study.

Potential benefits for the neonate are decreased rates of late onset sepsis, NEC, death.

**Expected outcomes, potential pitfalls, and alternative strategies*.*** We hypothesize that antibiotics will affect the microbial taxonomy, metabolic and inflammatory milieu of the developing gastrointestinal tract. We anticipate that non-pre-emptive use will not result in increased sepsis (non-inferiority). As in our previous research that related the development of the microbiome to NEC, sepsis, and preterm birth, we will evaluate microbial taxonomy[^25^](#_ENREF_25)^,^[^35-38^](#_ENREF_35), as well as more sophisticated metagenomics, metabolomics and inflammatory mediators (see below). We are well poised to analyze multi-omics data with our collaborator, Dr. Ana Conesa, who has specific expertise in multi-omic systems. **We anticipate finding significant and lasting differences in the microbiome, metabolome and host inflammatory response related to antibiotic usage. For example, along with altered microbiota at 30 days after birth, we will find altered metabolites with antibiotic treatment.** We are cognizant that these studies are not designed to determine specific mechanisms of disease pathogenesis, but instead, they are likely to provide robust associations in human preterm infants that will provide critical information for future studies of mechanisms as well as data that will provide strong impetus for prospective clinical trials to evaluate the necessity for the current widespread use of antibiotics.

**Aim 2** Test the effects of pre-emptive postnatal antibiotics on adverse outcomes in the neonate while in the NICU. We hypothesize that higher antibiotic use will not be associated with decreased early onset sepsis and will be associated with increased adverse outcomes including necrotizing enterocolitis, late onset sepsis, BPD, and mortality.

.

**Rationale**. Currently there are no studies correlating the developing microbiome, metabolome and inflammatory responses to subsequent adverse outcomes in the neonate in a prospective controlled manner. For aim 2, subjects enrolled in the Aim 1 studies will be followed in terms of their clinical status. The incidence of early onset sepsis (culture proven and suspected) will be evaluated and compared among and between the groups. Multivariable logistic regression will be conducted to determine independent relationships between initial empirical antibiotic therapy and study outcomes that control for birth weight, gestational age, race, prolonged premature rupture of membranes, days on mechanical ventilation, and the amount of breast milk received in the first 14 days after birth.

**Expected outcomes, potential pitfalls, and alternative strategies*.*** We anticipate there will be an increase in adverse outcomes that correspond to perturbations in the microbiome, metabolome and inflammatory markers in the group receiving antibiotics. There are several confounding factors including maternal antibiotics, mode of delivery, gestational age, and length of rupture of membranes that will be taken into account using multiple regressions.

**Specific Methodologies**

**Microbiota 16s rRNA, metagenomic sequencing, and analyses.** DNA, RNA and protein extraction, 16S rRNA amplication and sequencing, 16S rRNA analyses, metagenomic sequencing and genome assembly will be done as described previously. [^39^](#_ENREF_39) In our previous work where certain bacteria were negatively correlated with gestational age ^24^, we found that a single bacterial genus would dominate the microbiome (>50% of the population). If we see several samples dominated by a single genus, we will mine these samples for whole genomes using PacBio sequencing to get a broader view of their physiology as we have done in the past. [^39^](#_ENREF_39)^,^[^40^](#_ENREF_40) Where shotgun metagenomics are needed for other, more complex samples, either PacBio or Illumina sequencing will be done and the data analyzed using MG-RAST.[^41^](#_ENREF_41) Dr. Triplett’s lab is highly capable of culturing difficult to culture microbes, if needed.

**Basic diversity indices (Chao1, Shannon) & ordination methods (DCA with adonis).** Microbial diversity is assessed using Chao1, Shannon, and ordination methods implemented using the *phyloseq* package in R.[^42^](#_ENREF_42) Chao1 estimates the species richness for each sample, while the Shannon Index scores richness and abundance, though is not sufficient in assessing overall microbiome differences. Detrended Correspondence Analysis (DCA), a multivariate statistical method, will be applied to detect overall microbiome differences. Adonis methods were used to attribute additional variables’ contribution to microbial variance.

**Inflammatory markers.** Inflammatory markers will be analyzed by Dr. Nan Li using a combination of multiplex technologies using the BioRad Bio-Plex platform to obtain the comparative data from meconium samples. The markers evaluated include common markers of intestinal inflammation including calprotectin and S100A12, in addition to other markers such as IL-6, TNF, IL-10 and other cytokines and chemokines that may play a role in the inflammatory and/or anti-inflammatory process. These assays will be done on the stools of the babies. The data will be analyzed using direct comparisons of the 4 groups of infants using analysis of Variance and subsequent individual comparisons.

**Metabolomics.** The microbiome plays a prominent role in human metabolism because microbes produce harmful and beneficial small molecules such as vitamins, polyphenols, cholesterol and short chain fatty acids[^43^](#_ENREF_43). These analyses will be conducted at the CTSI Southeast Center for Integrated Metabolomics at the University of Florida: http://secim.ufl.edu/. Metabolites in stool as biomarkers of microbial-host metabolism and breast milk samples will be identified by state-of-the-art NMR and MS.

**Data integration.**

Since this research will also include a clinical trial, DHHS regulations to protect humans from research risks will be undertaken. This will include parental informed consent, IRB approval, and registration with ClinicalTrials.gov.

**Statistical Analysis.**

Participants will be randomized by block randomization using random block sizes of two and four.  The statistician on the study (Dr. Matthew Gurka) will create and store a SAS program with a fixed seed that randomizes the participants and can be reproduced if needed. The resulting randomization assignment file will be uploaded into the REDCap study database, where the randomization module will be used to by the study coordinator (Kelly Curry) or other study team member.  As successive patients are recruited and enrolled in the study, and found to be eligible for random assignment, Ms. Curry or other study team member will use the randomization module in REDCap to identify their random assignment, which will subsequently remain in the database.

For the outcomes measured weekly (e.g., principal component scores from the 16S rRNA analyses, diversity indices, etc.) as part of Aim 1, generalized linear mixed models will be utilized to compare trends over time between the two experimental groups. The appropriate link function will be used depending on the distribution of the outcome modeled (e.g., a Poisson model for the bacterial taxa). Such a modeling framework will provide estimates of clinically meaningful effects such as differences in means at a given time point (or mean changes over time), while also providing variance and correlation estimates needed to adequately power the next larger study if deemed warranted.

Aim 2 analyses will be comprised of time-to-event analyses comparing the two groups (Kaplan-Meier curves, log-rank tests, and Cox proportional hazards models), focusing on a composite adverse event variable that incorporates NEC, late onset sepsis, and/or death. Secondary exploratory analyses will look at each of the events individually. An intent-to-treat analysis will be utilized throughout when comparing the two groups. Stratification by gestational age and other possible covariates will be performed in an exploratory manner.

**Sample Size Considerations**. As this is a pilot study, the sample size decision of 440 was driven primarily by obtaining estimates of feasibility, such as the consent rate, consented mothers that progress in their pregnancies past 33 weeks gestation, and estimates of the clinical outcomes in Aim 2 [^44^](#_ENREF_44). Additional items of interest include the proportion in the control group who ultimately are treated with antibiotics. In these cases, n = 50 in either group would lead to a maximum two-sided 95% confidence interval half-width equal to 0.145 (when a given rate = 0.50), with more precise CI’s when the rate is in either direction. For example, if the observed rate of eventual antibiotic administration in the control group was 0.25, the 95% CI (using exact methods) would be (0.14, 0.39), providing sound data to plan a future trial. The above calculations also hold when examining the clinical outcomes in Aim 2, where we expect an adverse event rate of approximately 20-25%. The analysis plan will provide for statistical inference when comparing groups in with respect to the outcomes in both Aims 1 and 2; however with no prior reliable data there is no basis for a formal power analysis, and 50 per group will provide for sound estimates of variability and correlations, as well as inform whether a subsequent trial should focus on superiority or non-inferiority of not administering antibiotics in this population.

**Safety:** It is strongly suggested that all subjects have infection-screening labs done soon after delivery as per NICU standard of care to include CBC with differential, blood cultures, and C-reactive protein. Final determination about screening labs will be left to the judgment of the primary medical team. Subjects who are randomized to Group C, no antibiotics, have a small risk of developing an early onset infection. Adverse events for the study will include septicemia and death.

Common morbidities which will be recorded are NEC, SIP, IVH/PVL, ROP, CLD, BPD, and rates of sepsis.

DSMB is in place. The DSMB will meet after of 20 and 50 subjects have attained 32 weeks corrected age or discharge or earlier if there is any suggestion of greater than usual number of adverse events in patients enrolled in the study.

**Conflict of interest:**

The principal investigator and co-investigators have no conflicts of interest in this study.

**Timetable:** In year 1, 440 subjects are recruited into the trial, 100 of whom we anticipate randomizing. In year 2, we will complete the analyses, publish our results and design the next phase of this investigation.

**Summary:** At the end of this 2 year project, we will have effectively challenged existing dogma that nearly all preterm infants require at least a short course of intravenous antibiotics. We will have a better understanding of the risks versus benefits of early pre-emptive antibiotic use in preterm infants. We will have a better understanding of the effects of antibiotics routinely given to pregnant women and preterm infants on the developing intestinal and human milk microbiome, metabolome and inflammatory status. It is anticipated that correlations to common morbidities such as late onset sepsis and necrotizing enterocolitis will be found. These will provide targets for future mechanistic based studies of causality. A greater justification for larger randomized trials may also result.

**References**

1. Dethlefsen L, Huse S, Sogin ML, Relman DA. The pervasive effects of an antibiotic on the human gut microbiota, as revealed by deep 16S rRNA sequencing. PLoS biology 2008;6:e280.

2. Dethlefsen L, Relman DA. Incomplete recovery and individualized responses of the human distal gut microbiota to repeated antibiotic perturbation. Proc Natl Acad Sci U S A 2011;108 Suppl 1:4554-61.

3. Fouhy F, Guinane CM, Hussey S, et al. High-throughput sequencing reveals the incomplete, short-term recovery of infant gut microbiota following parenteral antibiotic treatment with ampicillin and gentamicin. Antimicrobial agents and chemotherapy 2012;56:5811-20.

4. Cotten CM, Taylor S, Stoll B, et al. Prolonged duration of initial empirical antibiotic treatment is associated with increased rates of necrotizing enterocolitis and death for extremely low birth weight infants. Pediatrics 2009;123:58-66.

5. Alexander VN, Northrup V, Bizzarro MJ. Antibiotic exposure in the newborn intensive care unit and the risk of necrotizing enterocolitis. J Pediatr 2011;159:392-7.

6. Blaser M. Antibiotic overuse: Stop the killing of beneficial bacteria. Nature 2011;476:393-4.

7. Tripathi N, Cotten CM, Smith PB. Antibiotic use and misuse in the neonatal intensive care unit. Clin Perinatol 2012;39:61-8.

8. Kuppala VS, Meinzen-Derr J, Morrow AL, Schibler KR. Prolonged initial empirical antibiotic treatment is associated with adverse outcomes in premature infants. J Pediatr 2011;159:720-5.

9. Maynard CL, Elson CO, Hatton RD, Weaver CT. Reciprocal interactions of the intestinal microbiota and immune system. Nature 2012;489:231-41.

10. Deshmukh HS, Liu Y, Menkiti OR, et al. The microbiota regulates neutrophil homeostasis and host resistance to Escherichia coli K1 sepsis in neonatal mice. Nat Med 2014;20:524-30.

11. Lamouse-Smith ES, Tzeng A, Starnbach MN. The intestinal flora is required to support antibody responses to systemic immunization in infant and germ free mice. PLoS One 2011;6:e27662.

12. Clark RH, Bloom BT, Spitzer AR, Gerstmann DR. Reported medication use in the neonatal intensive care unit: data from a large national data set. Pediatrics 2006;117:1979-87.

13. Stoll BJ, Gordon T, Korones SB, et al. Early-onset sepsis in very low birth weight neonates: a report from the National Institute of Child Health and Human Development Neonatal Research Network. J Pediatr 1996;129:72-80.

14. Goldenberg RL, Andrews WW, Goepfert AR, et al. The Alabama Preterm Birth Study: umbilical cord blood Ureaplasma urealyticum and Mycoplasma hominis cultures in very preterm newborn infants. Am J Obstet Gynecol 2008;198:43 e1-5.

15. Schulman J, Dimand RJ, Lee HC, Duenas GV, Bennett MV, Gould JB. Neonatal intensive care unit antibiotic use. Pediatrics 2015;135:826-33.

16. Thinkhamrop J, Hofmeyr GJ, Adetoro O, Lumbiganon P, Ota E. Antibiotic prophylaxis during the second and third trimester to reduce adverse pregnancy outcomes and morbidity. The Cochrane database of systematic reviews 2015;1:CD002250.

17. Schrag SJ, Verani JR. Intrapartum antibiotic prophylaxis for the prevention of perinatal group B streptococcal disease: experience in the United States and implications for a potential group B streptococcal vaccine. Vaccine 2013;31 Suppl 4:D20-6.

18. Dardas M, Gill SR, Grier A, et al. The impact of postnatal antibiotics on the preterm intestinal microbiome. Pediatr Res 2014;76:150-8.

19. Arboleya S, Sanchez B, Milani C, et al. Intestinal microbiota development in preterm neonates and effect of perinatal antibiotics. J Pediatr 2015;166:538-44.

20. Fricke WF. The more the merrier? Reduced fecal microbiota diversity in preterm infants treated with antibiotics. J Pediatr 2014;165:8-10.

21. DiGiulio DB. Prematurity and perinatal antibiotics: a tale of two factors influencing development of the neonatal gut microbiota. J Pediatr 2015;166:515-7.

22. Tzialla C, Borghesi A, Serra G, Stronati M, Corsello G. Antimicrobial therapy in neonatal intensive care unit. Italian journal of pediatrics 2015;41:27.

23. Neu J, Walker WA. Necrotizing enterocolitis. N Engl J Med 2011;364:255-64.

24. Torrazza RM, Ukhanova M, Wang X, et al. Intestinal microbial ecology and environmental factors affecting necrotizing enterocolitis. PloS one 2013;8:e83304.

25. Mai V, Torrazza RM, Ukhanova M, et al. Distortions in development of intestinal microbiota associated with late onset sepsis in preterm infants. PLoS One 2013;8:e52876.

26. Mai V, Young CM, Ukhanova M, et al. Fecal microbiota in premature infants prior to necrotizing enterocolitis. PloS one 2011;6:e20647.

27. Wang Y, Hoenig JD, Malin KJ, et al. 16S rRNA gene-based analysis of fecal microbiota from preterm infants with and without necrotizing enterocolitis. ISME J 2009;3:944-54.

28. Carl MA, Ndao IM, Springman AC, et al. Sepsis from the gut: the enteric habitat of bacteria that cause late-onset neonatal bloodstream infections. Clin Infect Dis 2014;58:1211-8.

29. Greenwood C, Morrow AL, Lagomarcino AJ, et al. Early Empiric Antibiotic Use in Preterm Infants Is Associated with Lower Bacterial Diversity and Higher Relative Abundance of Enterobacter. J Pediatr 2014.

30. Mshvildadze M, Neu J, Schuster J, Theriaque D, Li N, Mai V. Intestinal microbial ecology in premature infants assessed with non-culture-based techniques. J Pediatr 2010;156:20-5.

31. Madan JC, Farzan SF, Hibberd PL, Karagas MR. Normal neonatal microbiome variation in relation to environmental factors, infection and allergy. Current opinion in pediatrics 2012;24:753-9.

32. Flenady V, Hawley G, Stock OM, Kenyon S, Badawi N. Prophylactic antibiotics for inhibiting preterm labour with intact membranes. The Cochrane database of systematic reviews 2013;12:CD000246.

33. Fritz JV, Desai MS, Shah P, Schneider JG, Wilmes P. From meta-omics to causality: experimental models for human microbiome research. Microbiome 2013;1:14.

34. Myatt L, Eschenbach DA, Lye SJ, et al. A standardized template for clinical studies in preterm birth. Reproductive sciences (Thousand Oaks, Calif) 2012;19:474-82.

35. Mshvildadze M, Neu J, Schuster J, Theriaque D, Li N, Mai V. Microbial Ecology of the premature infant using non Culture ased techniques. 2009.

36. Mai V, Young CM, Ukhanova M, et al. Fecal microbiota in premature infants prior to necrotizing enterocolitis. PLoS One 2011;6:e20647.

37. Murgas Torrazza R, Neu J. The developing intestinal microbiome and its relationship to health and disease in the neonate. J Perinatol 2011;31:S29-34.

38. Ardissone AN, de la Cruz DM, Davis-Richardson AG, et al. Meconium microbiome analysis identifies bacteria correlated with premature birth. PLoS One 2014;9:e90784.

39. Davis-Richardson AG, Ardissone AN, Dias R, et al. Bacteroides dorei dominates gut microbiome prior to autoimmunity in Finnish children at high risk for type 1 diabetes. Frontiers in microbiology 2014;5:678.

40. Leonard MT, Davis-Richardson AG, Ardissone AN, et al. The methylome of the gut microbiome: disparate Dam methylation patterns in intestinal Bacteroides dorei. Frontiers in microbiology 2014;5:361.

41. Meyer F, Paarmann D, D'Souza M, et al. The metagenomics RAST server - a public resource for the automatic phylogenetic and functional analysis of metagenomes. BMC bioinformatics 2008;9:386.

42. McMurdie PJ, Holmes S. phyloseq: an R package for reproducible interactive analysis and graphics of microbiome census data. PloS one 2013;8:e61217.

43. Nicholson JK, Holmes E, Kinross J, et al. Host-gut microbiota metabolic interactions. Science 2012;336:1262-7.

44. Moore CG, Carter RE, Nietert PJ, Stewart PW. Recommendations for planning pilot studies in clinical and translational research. Clinical and translational science 2011;4:332-7.


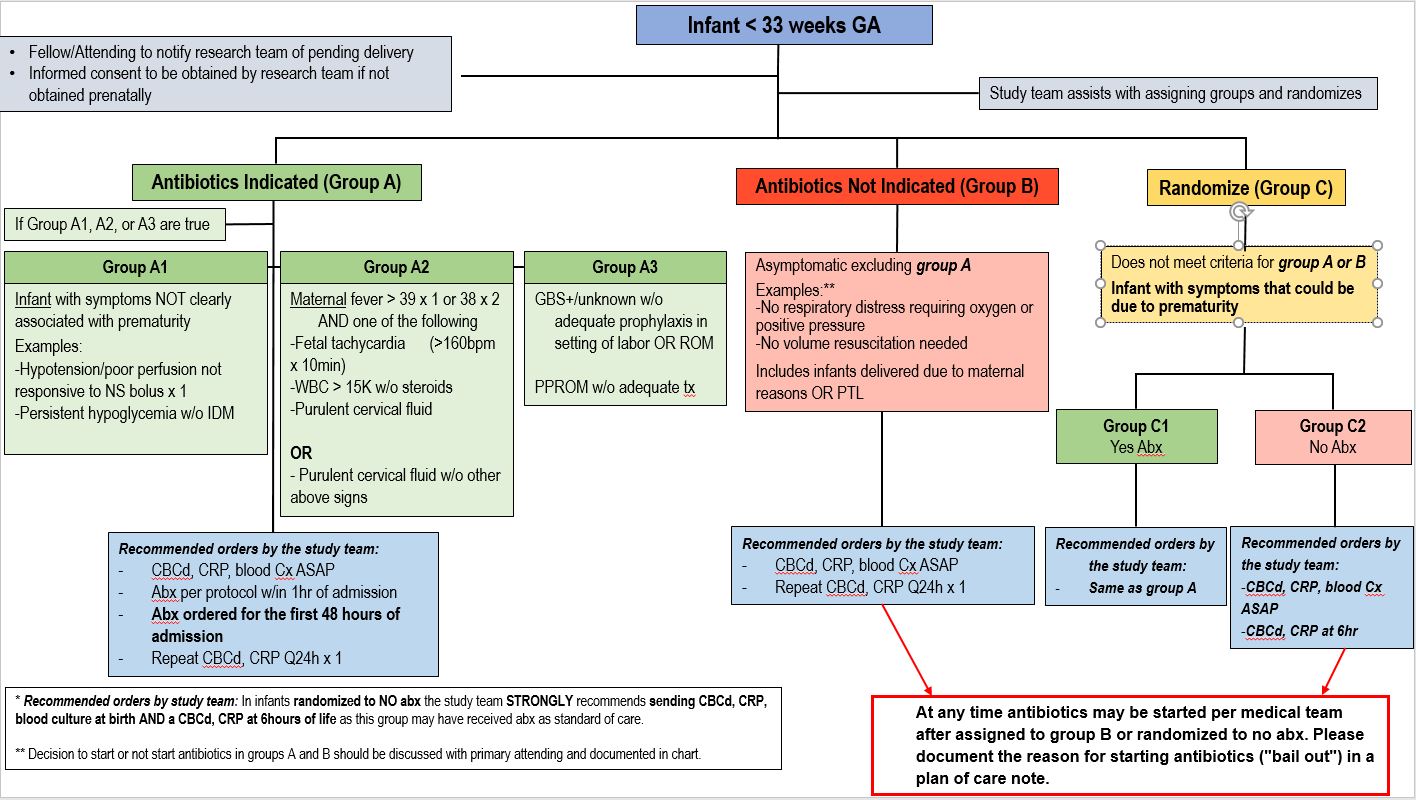

Supplement: Supplementary file 3 — Supplementary Information. [file 41598_2021_80982_MOESM3_ESM.docx]
